# A homeostatic clock sets daughter centriole size in flies

**DOI:** 10.1083/jcb.201801014

**Published:** 2018-04-02

**Authors:** Mustafa G. Aydogan, Alan Wainman, Saroj Saurya, Thomas L. Steinacker, Anna Caballe, Zsofia A. Novak, Janina Baumbach, Nadine Muschalik, Jordan W. Raff

**Affiliations:** 1 Sir William Dunn School of Pathology, University of Oxford, Oxford, England, UK; 2 Micron Oxford Advanced Bioimaging Unit, Department of Biochemistry, University of Oxford, Oxford, England, UK

## Abstract

Centrioles are highly structured organelles whose size is remarkably consistent within any given cell type. New centrioles are born when Polo-like kinase 4 (Plk4) recruits Ana2/STIL and Sas-6 to the side of an existing “mother” centriole. These two proteins then assemble into a cartwheel, which grows outwards to form the structural core of a new daughter. Here, we show that in early *Drosophila melanogaster* embryos, daughter centrioles grow at a linear rate during early S-phase and abruptly stop growing when they reach their correct size in mid- to late S-phase. Unexpectedly, the cartwheel grows from its proximal end, and Plk4 determines both the rate and period of centriole growth: the more active the centriolar Plk4, the faster centrioles grow, but the faster centriolar Plk4 is inactivated and growth ceases. Thus, Plk4 functions as a homeostatic clock, establishing an inverse relationship between growth rate and period to ensure that daughter centrioles grow to the correct size.

## Abstract (precis)

Centrioles are highly structured organelles of consistent size across cell types. Aydogan et al. show that, in early *Drosophila* embryos, Plk4 functions as a homeostatic clock, establishing an inverse relationship between growth rate and period to ensure that daughter centrioles grow to the correct size.

## Introduction

How organelles grow to the right size is a fundamental problem in cell biology (Marshall, 2016). For many organelles, however, this question is difficult to address: the number and distribution of an organelle within a cell can vary, and it can also be difficult to determine whether an organelle’s surface area, volume, or perhaps the amount of a limiting component, best defines its size. Centrioles are highly structured organelles that form centrosomes and cilia (Nigg and Raff, 2009). Their length can vary by an order of magnitude between different species and tissues but is very consistent within a given cell type. Centrioles are potentially an attractive system with which to study organelle size control (Goehring and Hyman, 2012; Marshall, 2016), as their numbers are precisely regulated: most cells are born with a single centriole pair that is duplicated once per cell cycle, when a single daughter centriole grows outwards from each mother centriole during S-phase. Moreover, the highly ordered structure of the centriole means that the complex 3D question of organelle size control can be simplified to a 1D question of daughter centriole length control.

Much progress has been made recently in understanding the molecular mechanisms of centriole duplication (Fırat-Karalar and Stearns, 2014; Arquint and Nigg, 2016; Banterle and Gönczy, 2017). Polo-like kinase 4 (Plk4) initiates duplication and is first recruited in a ring surrounding the mother centriole; this ring ultimately resolves into a single “dot” that marks the site of daughter centriole assembly (Sonnen et al., 2012; Kim et al., 2013; Ohta et al., 2014). Plk4 recruits and phosphorylates Ana2/STIL, which helps recruit Sas-6 to initiate the assembly of the ninefold-symmetric cartwheel that forms the structural backbone of the growing daughter centriole (Kitagawa et al., 2011; van Breugel et al., 2011; Dzhindzhev et al., 2014; Ohta et al., 2014; Kratz et al., 2015). How Plk4 is ultimately localized to a single site on the side of the mother is unclear, but Plk4 can dimerize and autophosphorylate itself in trans to trigger its own destruction (Rogers et al., 2009; Guderian et al., 2010; Holland et al., 2010; Cunha-Ferreira et al., 2013). In addition, binding to Ana2/STIL activates Plk4’s kinase activity (Moyer et al., 2015) and also appears to stabilize Plk4 (Ohta et al., 2014; Arquint et al., 2015). Thus, the binding of Plk4 to Ana2/STIL at a single site on the side of the mother could activate and protect the kinase at this site, whereas the remaining Plk4 around the mother centriole is degraded (Ohta et al., 2014; Moyer et al., 2015; Arquint and Nigg, 2016; Banterle and Gönczy, 2017).

Although these studies provide important insight into how mother centrioles grow only a single daughter, the question of how daughter centrioles subsequently grow to the correct length has been difficult to address (Winey and O’Toole, 2014). This is in part because centrioles are small structures (usually 100–500 nm in length), making it hard to directly monitor the kinetics of centriole growth. Also, cells usually only assemble two daughter centrioles per cell cycle, and this makes it difficult to measure centriole growth in a quantitative manner. The early *Drosophila melanogaster* embryo is an established model for studying centriole and centrosome assembly (Lattao et al., 2017), and it is potentially an attractive system for measuring the kinetics of daughter centriole growth. First, it is a multinucleated single cell (a syncytium) that undergoes 13 rounds of nearly synchronous, rapid nuclear divisions (Foe and Alberts, 1983). During nuclear cycles 10–14, the majority of nuclei (and their associated centrioles) form a monolayer at the cortex, allowing the simultaneous observation of many centrioles as they rapidly and synchronously progress through repeated rounds of S-phase and mitoses without intervening gap phases (Foe and Alberts, 1983). Second, centrioles in flies are structurally simpler than those in vertebrates (Callaini and Riparbelli, 1990; Moritz et al., 1995; Callaini et al., 1997). All centrioles start to assemble around the cartwheel in S-phase, but vertebrate centrioles often exhibit a second phase of growth during G2/M, when the centriolar microtubules (MTs) extend past the cartwheel (Kuriyama and Borisy, 1981; Chrétien et al., 1997). Fly centrioles usually do not exhibit this second phase of growth, so the centrioles are relatively short, and the cartwheel extends throughout the length of the daughter centriole (González et al., 1998; Lattao et al., 2017). We reasoned, therefore, that the fluorescence incorporation of the cartwheel components Sas-6-GFP or Ana2-GFP could potentially be used as a proxy to measure daughter centriole length in *D. melanogaster* embryos.

We show here that this is the case, and we provide the first quantitative description of the kinetics of daughter centriole growth in a living cell. Our findings reveal an unexpected inverse relationship between the centriole growth rate and growth period: in embryos where daughter centrioles tend to grow slowly, they tend to grow for a longer period. Surprisingly, Plk4 influences both the centriole growth rate and growth period and helps coordinate the inverse relationship between them. Thus, Plk4 functions as a homeostatic clock that helps to ensure daughter centrioles grow to the correct size in fly embryos.

## Results

### The cartwheel protein Sas-6 is incorporated irreversibly into growing daughter centrioles

**Figure 1.**
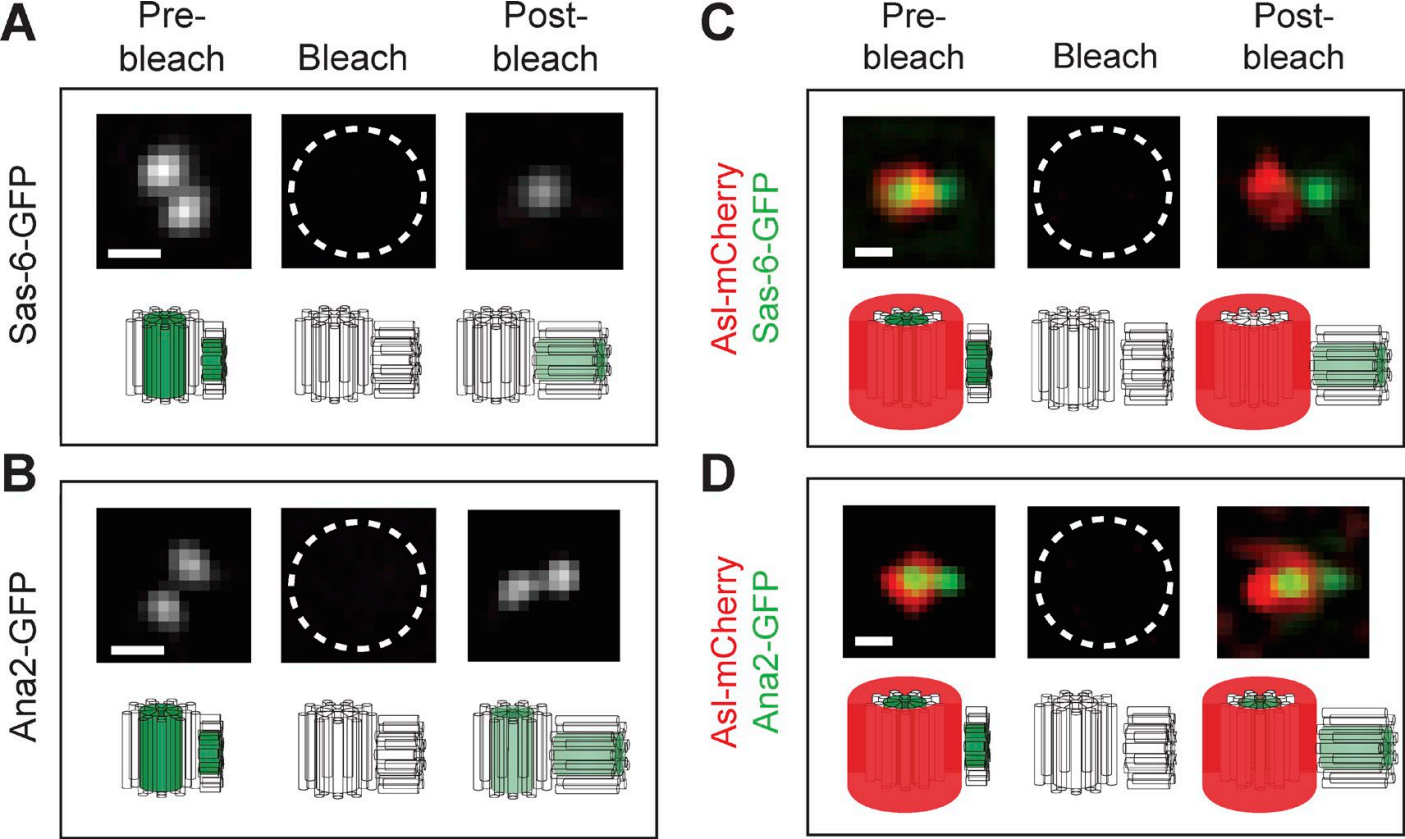
**Sas-6-GFP is incorporated irreversibly into the growing daughter centriole. (A and B)** Micrographs show a 3D-SIM-FRAP analysis of Sas-6-GFP (A) or Ana2-GFP (B) dynamics. A 3D-SIM image of a centriole pair was acquired in early S-phase (Pre-bleach); the centrioles were then photobleached (Bleach), and a post-bleach image was acquired 5–10 min later. **(C and D)** The same image acquisition protocol was followed in embryos that simultaneously express the mother centriole marker Asl-mCherry along with either Sas-6-GFP (C) or Ana2-GFP (D; note that bleaching the Sas-6- or Ana2-GFP, as an unintended consequence, also bleaches the Asl-mCherry fluorescence). Asl-mCherry fluorescence rapidly recovers at the mother centriole, as reported previously (Novak et al., 2014, 2016), as does Ana2-GFP fluorescence, indicating that both proteins turn over at the fully grown mother centrioles. In contrast, Sas-6-GFP does not detectably recover at the mother centriole, but does recover at the daughter. This suggests that once Sas-6-GFP molecules are incorporated into the cartwheel structure they do not turn over, at least not over the time course of these experiments. The underlying schematics illustrate our interpretation of the behavior of the GFP and mCherry fusions. Bars, 0.2 µm. *n* = 4 embryos; *n* = 15 centriole pairs for each protein in each experiment.

To establish an assay to monitor centriole growth kinetics, we tested whether Sas-6 and/or Ana2 are stably incorporated into growing daughter centrioles. We generated transgenic lines expressing either Sas-6-GFP or Ana2-GFP under the control of their own promoters and in their respective mutant backgrounds (Fig. S1, A and B). These fusion proteins significantly rescued the severe uncoordinated phenotype (caused by the lack of centrioles) in their respective mutant backgrounds (Fig. S1, C and D; and Videos 1 and 2). 3D structured illumination microscopy (SIM) FRAP (Conduit et al., 2015) revealed that Sas-6-GFP and Ana2-GFP were both incorporated into growing daughter centrioles during S-phase; but, whereas Ana2-GFP turned over at the mother centrioles, Sas-6-GFP did not (Fig. 1). Thus, during the time course of these experiments, Sas-6-GFP is incorporated exclusively and irreversibly into the growing daughter centriole, making it a suitable marker to monitor daughter centriole growth kinetics.

### Centrioles grow approximately linearly during early S-phase but abruptly stop growing in mid-late S-phase

**Figure 2.**
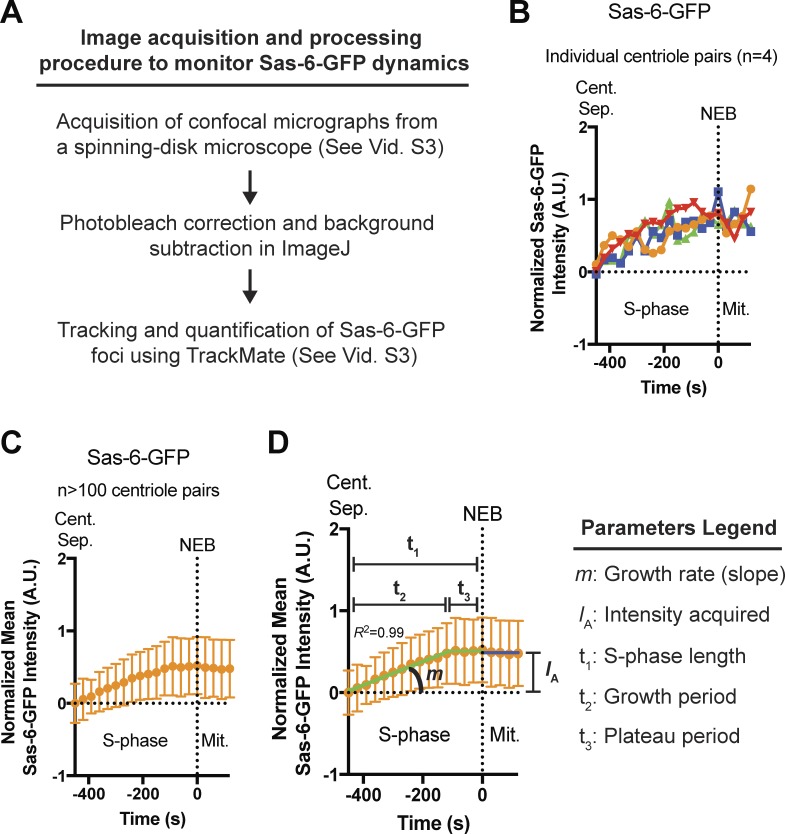
**Measuring the parameters of daughter centriole growth. (A)** Schematic summary of the Sas-6-GFP image acquisition and processing procedure used to monitor Sas-6-GFP dynamics over time (see also Video 3). **(B)** Graph shows the Sas-6-GFP fluorescence intensity over time measured from four different centriole-pair tracks during nuclear cycle 12 (A.U., arbitrary units; Cent. Sep., time centriole pair separation first detected at the start of S-phase; NEB, nuclear envelope breakdown). Note that when Sas-6-GFP is imaged using spinning-disk confocal microscopy, the mother and growing daughter centrioles cannot be resolved, and so they appear as a single fluorescent focus. Therefore, to measure the growth of the daughter centriole, all centriolar Sas-6-GFP intensities were normalized by subtracting the mean initial intensity of all the mother centrioles in that embryo at the start of S-phase (so that the mean Sas-6-GFP fluorescence at the start of S-phase is 0 in every embryo). **(C)** Same as in B, but the mean has been taken of the fluorescence intensity of >100 centriole pairs from the same embryo. **(D)** The S-phase (green line) and M-phase (dark blue line) data from C were fitted by regression analysis (see Fig. S2 for a summary of the models tested). *R^2^* is used as a measure of goodness-of-fit. From this model, several parameters of centriole growth were measured for each individual embryo (as indicated in the figure). Data are represented as mean ± SD.

**Figure 3.**
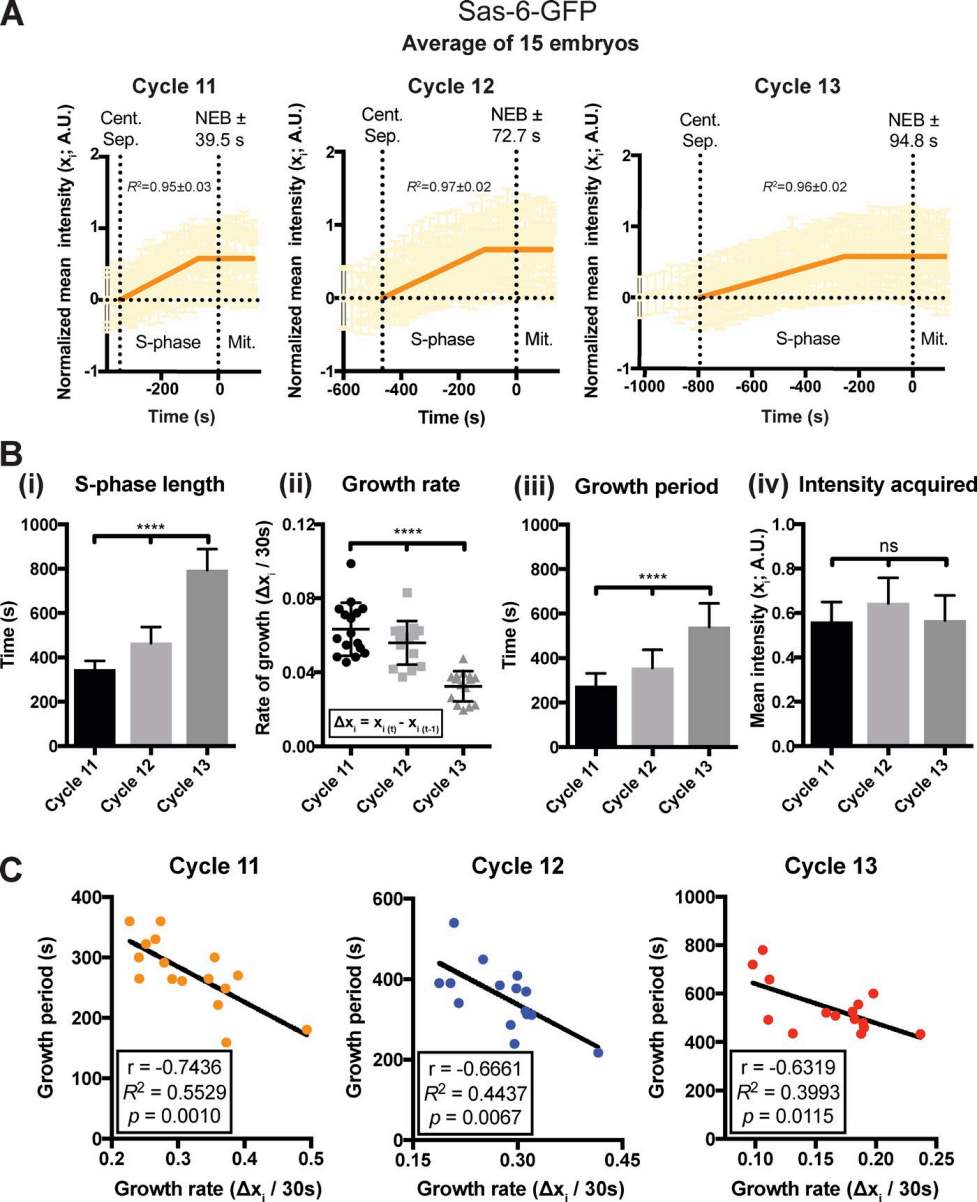
**An inverse relationship between the centriole growth rate and period sets daughter centriole size. (A)** Graphs show a “mean” centriolar Sas-6-GFP incorporation profile (orange line; *n* ≥ 15 embryos) for embryos at nuclear cycles 11, 12, or 13. The underlying data from each embryo in this and all subsequent “mean” graphs are shown in partial opacity. The ± associated with NEB represents the SD from the mean value. A.U., arbitrary units. **(B)** Bar charts quantify several parameters of centriole growth derived from the mean Sas-6-GFP incorporation profiles shown in A. Statistical significance was assessed using either an ordinary one-way ANOVA test (for Gaussian-distributed data) or a Kruskal–Wallis test (****, P < 0.0001; ns, not significant). Data are presented as mean ± SD. **(C)** Graphs show the inverse correlation between the centriole growth rate and growth period in individual embryos during each nuclear cycle. The data were extracted from the data shown in A. Correlation strength was examined using Pearson’s correlation coefficient (*r* < 0.40 weak; 0.40 < *r* < 0.60 moderate; *r* > 0.6 strong), and the significance of correlation was determined by the p-value (P < 0.05). *n* ≥ 15 embryos for each cell cycle; *n* = 96, 174, and 326 centrioles (mean) per embryo in each cycle, respectively.

We analyzed the kinetics of Sas-6-GFP incorporation into daughter centrioles in individual *Sas-6* mutant embryos during nuclear cycle 12, using spinning-disk confocal microscopy to track Sas-6-GFP foci (comprising the mother and growing daughter centriole, which cannot be resolved on this microscope system) from early S-phase, when the two separating mother centrioles first become visible as distinct entities (Fig. 2 A and Video 3). When measured at a single centriole pair, Sas-6-GFP levels tended to increase over time, but the data were noisy and it was difficult to discern a consistent pattern (Fig. 2 B). When measurements from >100 centriole pairs from the same embryo were averaged, however, a clear pattern emerged (Fig. 2 C). We used regression analysis to fit the mean data from each embryo to several different growth models (Figs. S2 and S3; see Materials and methods): centriole growth was usually best described by a period of linear growth in early-S-phase that plateaued in mid-to-late S-phase, presumably when the growing centrioles had reached their correct size, and this plateau continued into mitosis. A similar pattern of “linear growth in S-phase followed by a plateau in mitosis” has been reported previously for GFP:SAS-6 in the early worm embryo, suggesting that the dynamics of Sas-6 incorporation are likely to be conserved (Dammermann et al., 2008). From the fitted data, we extracted several growth parameters (Fig. 2 D) and used these to generate an average centriole growth profile derived from ∼1,100 individual centrioles tracked throughout S-phase in 15 individual embryos (Figs. 3 and S3).

### Centriole length is regulated by a homeostatic inverse relationship between growth rate and period

We wondered whether the parameters of centriole growth would change as S-phase length gradually increased during embryogenesis (Foe and Alberts, 1983), so we compared the parameters of centriole growth during nuclear cycles 11–13 (Fig. 3 A). As S-phase length increased (Fig. 3 Bi), the centriole growth rate slowed (Fig. 3 Bii), but this was precisely compensated for by an increase in the growth period (Fig. 3 Biii), and thus daughter centrioles ultimately incorporated the same amount of Sas-6-GFP during each nuclear cycle, indicating that they had grown to the same length (Fig. 3 Biv). Interestingly, a similar inverse relationship between growth rate and growth period was observed between embryos that were at the same nuclear cycle (Fig. 3 C). These observations raised the intriguing possibility that centriole growth in these embryos is regulated by a homeostatic mechanism, whereby an inverse relationship between the growth rate and period ensures that daughter centrioles grow to a consistent size.

### The rate and period of centriole growth are not simply determined by S-phase length

**Figure 4.**
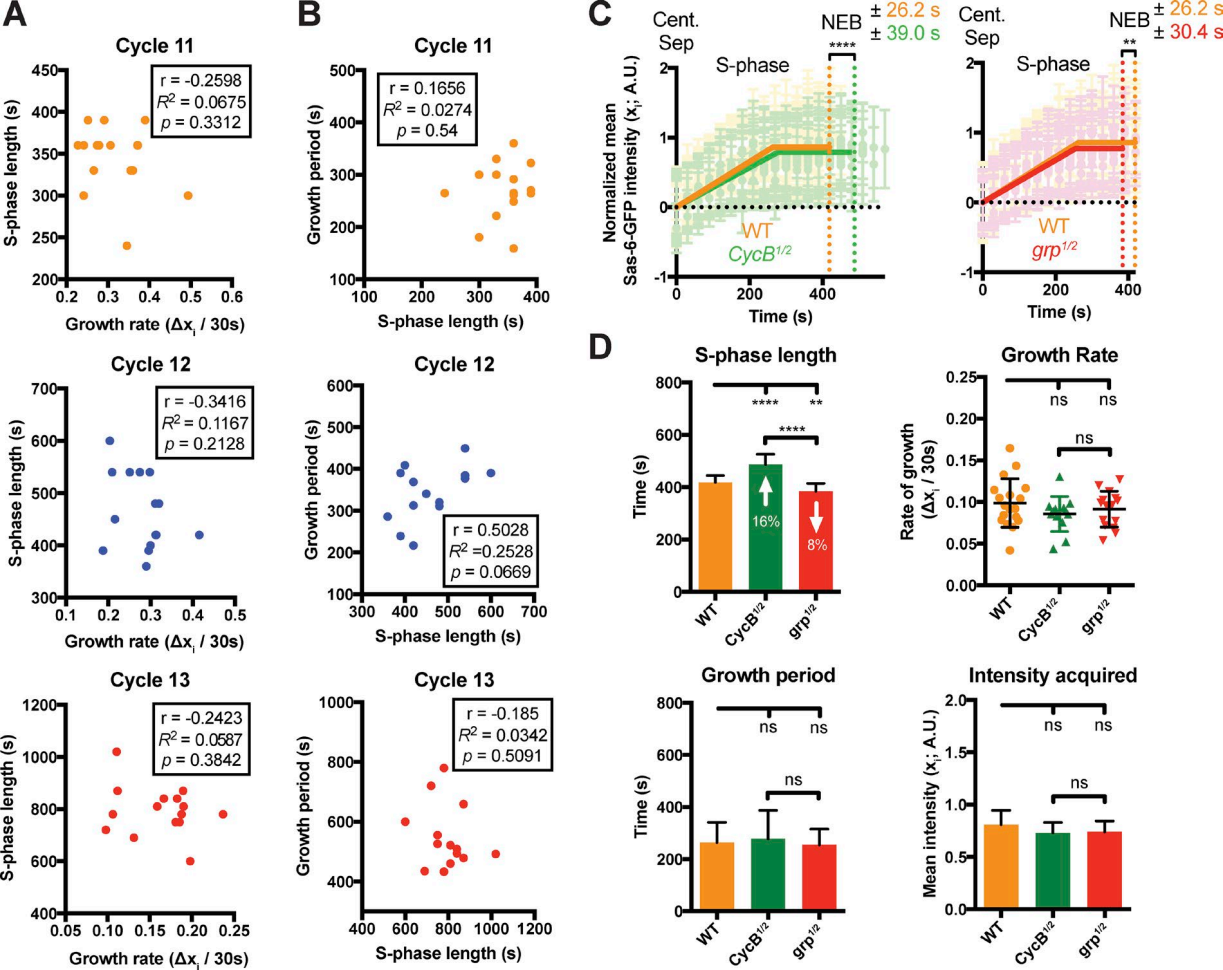
**The rate and period of daughter centriole growth are not directly determined by the length of S-phase. (A and B)** Graphs show the lack of a consistent correlation between the length of S-phase and either the centriole growth rate (A) or growth period (B), in each nuclear cycle. The data were extracted from the data shown in Fig. 3 A and analyzed as described in Fig. 3 C. **(C and D)** Graphs and charts illustrate and quantify centriole growth parameters in WT embryos and embryos in which either the genetic dose of Cyclin B has been halved to increase the length of S-phase (Ji et al., 2004; *CycB^1/2^* embryos) or the genetic dose of the S-phase checkpoint protein grapes (Chk1 in vertebrates) has been halved to decrease the length of S-phase (Sibon et al., 1997; *grp^1/2^* embryos); ≥13 embryos were analyzed in each case. Although S-phase is ∼24% longer in *CycB^1/2^* embryos compared with *grp^1/2^* embryos, the parameters of centriole growth are essentially the same in all three types of embryos. Statistical significance was assessed using either an unpaired *t* test with Welch’s correction (for Gaussian-distributed data) or an unpaired Mann–Whitney test (**, P < 0.01; ****, P < 0.0001; ns, not significant). A.U., arbitrary units. *n* ≥ 13 embryos for each group; *n* = 184, 140, and 122 centrioles (mean) from WT, *CycB^1/2^*, and *grp^1/2^* embryos, respectively. Data are presented as mean ± SD.

Perhaps surprisingly, there was no significant correlation between the length of S-phase and the centriole growth rate or growth period in embryos at the same nuclear cycle (Fig. 4, A and B). To more directly test whether S-phase length influences the parameters of centriole growth, we genetically manipulated S-phase length by either halving the genetic dose of cyclin B, which leads to an increase in S-phase length (Ji et al., 2004), or by halving the genetic dose of the DNA checkpoint protein *Chk1* (*grapes* in flies), which leads to a decrease in S-phase length (Sibon et al., 1997; Fig. 4, C and D). Although there was a ∼24% difference in the length of S-phase between these two sets of embryos, the parameters of centriole growth were not significantly altered (Fig. 4 D), demonstrating that S-phase length does not directly determine the centriole growth rate or period.

### Plk4 influences the rate and period of daughter centriole growth

**Figure 5.**
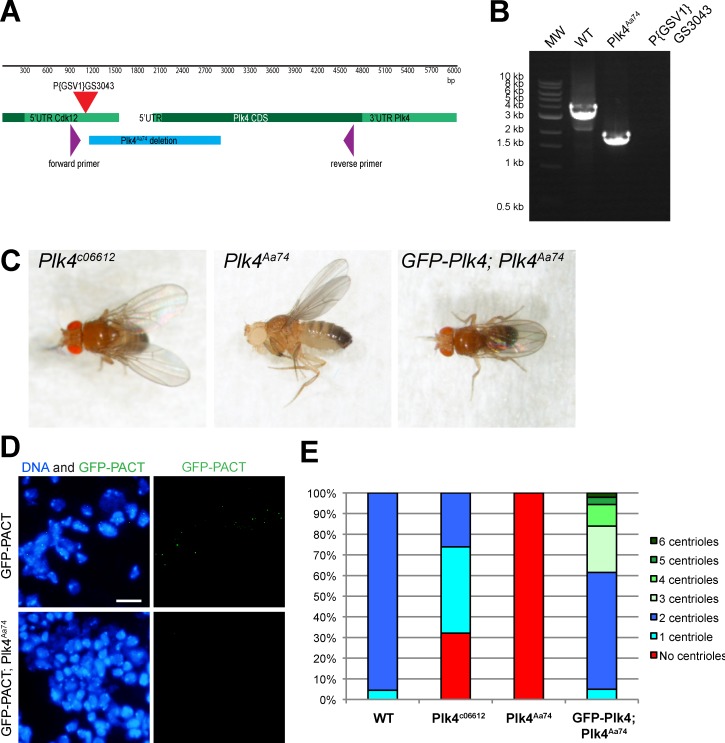
**Generation of the *Plk4^Aa74^* mutant allele.** Previous studies of *Plk4* have used the weak hypomorphic *Plk4^c06612^* allele (Bettencourt-Dias et al., 2005), so we generated a stronger allele (*Plk4^Aa74^*) by imprecise excision of the *Plk4^c06612^* P-element. **(A)** The schematic shows the *PLK4* genomic region, indicating the coding sequence (CDS; dark green), the UTRs (light green), the position of the original P{GSV1}GS3043 P-element insertion (red triangle), the position of the primers used to screen for imprecise excision of the P-element (purple), and the position of the Aa74 deletion (which deletes essentially the entire protein kinase domain; blue). **(B)** DNA gel shows the PCR products observed when these primers were used to amplify DNA from either WT flies or the homozygous *Plk4^Aa74^* deletion flies. Sequencing of the PCR products confirmed deletion of the 1,751-bp region indicated in A. MW stands for molecular weight marker as a unit label. **(C)** Photographs of adult flies taken without anesthesia (see also Video 4): the original *Plk4^c06612^* mutant flies can maintain a normal body posture and walk in a partially uncoordinated manner; *Plk4^Aa74^* flies are highly uncoordinated and appear to lack all proprioception, as is typical of flies lacking centrioles (Basto et al., 2006); GFP-Plk4 expression rescues the uncoordinated phenotype of *Plk4^Aa74^* mutant flies. **(D)** Micrographs show WT or *Plk4^Aa74^* third-instar larval brains, with DNA stained with Hoechst (blue) and centrioles revealed with GFP-PACT staining (green). Bar, 10 µm. **(E)** Bar chart quantifies the number of centrioles in mitotic larval brain cells of various genotypes (as indicated). *n* = 12 brains; *n* = 264 cells *in toto* for WT; 5 brains, 237 cells for *Plk4^c06612^*; 7 brains, 355 cells for *Plk4^Aa74^*; 4 brains, 143 cells for GFP-Plk4; *Plk4^Aa74^*. Collectively, these data indicate that the *Plk4^Aa74^* allele behaves as a null or strong hypomorph.

**Figure 6.**
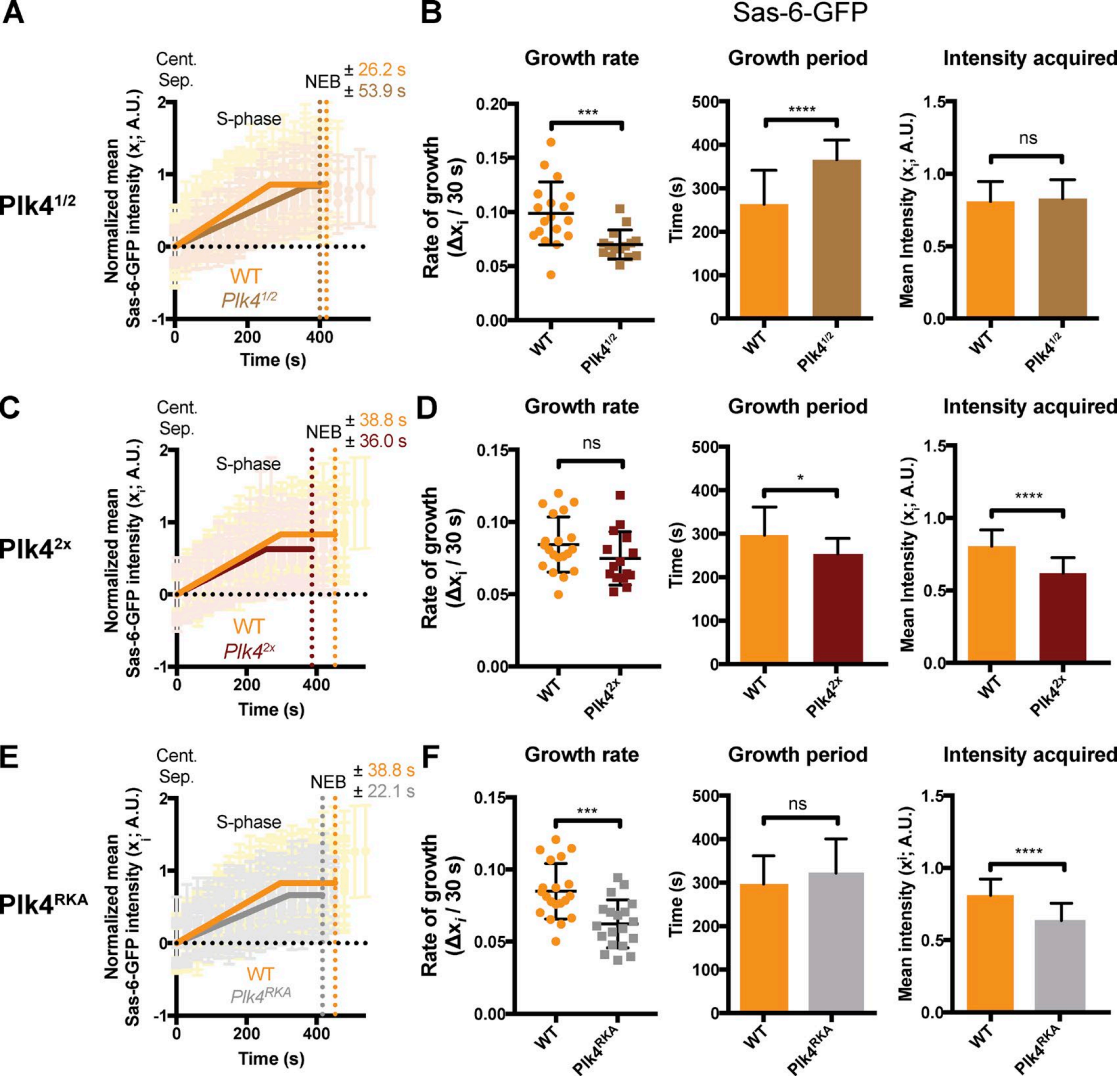
**Plk4 levels influence the rate and period of centriole growth. (A)** Graphs compare the mean Sas-6-GFP incorporation profile during nuclear cycle 12 in WT embryos (orange) and *Plk4^1/2^* embryos (brown). **(B)** Charts quantify parameters of centriole growth derived from the Sas-6-GFP incorporation profiles in A. **(C–F)** Graphs and charts show the same analysis for *Plk4^2x^* (dark brown) and *Plk4^RKA^* embryos (gray). Data are represented as mean ± SD (*n* ≥ 15 embryos for each group). Statistical significance was assessed using either an unpaired *t* test with Welch’s correction (for Gaussian-distributed data) or an unpaired Mann–Whitney test (*, P < 0.05; ***, P < 0.001; ****, P < 0.0001; ns, not significant). A.U., arbitrary units. *n* ≥ 15 embryos for each group; *n* = 92, 84, 98, and 39 centrioles (mean) from each WT, *Plk4^1/2^*, *Plk4^2x^*, and *Plk4^RKA^* embryo, respectively.

Plk4 determines the site of daughter centriole assembly (Arquint and Nigg, 2016; Banterle and Gönczy, 2017), so we wondered whether it might also influence the parameters of daughter centriole growth. We generated a likely null allele of *Plk4* (Fig. 5 and Video 4), and then monitored Sas-6-GFP incorporation in early embryos in which we halved the genetic dose of *Plk4* (hereafter, *Plk4^1/2^* embryos; Fig. 6 A). *Plk4^1/2^* embryos hatched at normal rates (Fig. S4), and we observed no centriole duplication defects in our analysis of >1,300 duplication events. Compared with WT embryos, however, the daughter centrioles in *Plk4^1/2^* embryos grew more slowly, but for a longer period of time; as a result the centrioles ultimately grew to their normal size (Fig. 6, A and B). These findings support our conclusion that there is a homeostatic inverse relationship between the growth rate and growth period that ensures daughter centrioles grow to a consistent size. Moreover, they suggest that Plk4 influences both the rate and period of daughter centriole growth, and so helps to establish this inverse relationship.

We next tested the effect of either doubling the genetic dosage of *Plk4* (*Plk4^2X^*) or expressing a previously described mutated form of Plk4 with reduced kinase activity (Holland et al., 2010; Zitouni et al., 2016; *Plk4^RKA^*) in a WT background (Fig. 6, C–F). Both sets of embryos hatched at normal rates (Fig. S4), and we observed no centriole duplication defects in our analysis of >1,400 duplication events in each genotype. Surprisingly, however, both perturbations resulted in a significant decrease in daughter centriole size, but for different reasons: in *Plk4^2X^* embryos, the centrioles grew at a normal rate but for a shorter period (Fig. 6, C and D), whereas in *Plk4^RKA^* embryos, the centrioles grew at a slower rate but for a normal period (Fig. 6, E and F). Thus, Plk4 appears to influence the daughter centriole growth rate and growth period independently, with the extra Plk4 influencing the growth period and reduced Plk4 kinase activity influencing the growth rate.

### A second assay to measure the parameters of daughter centriole growth

**Figure 7.**
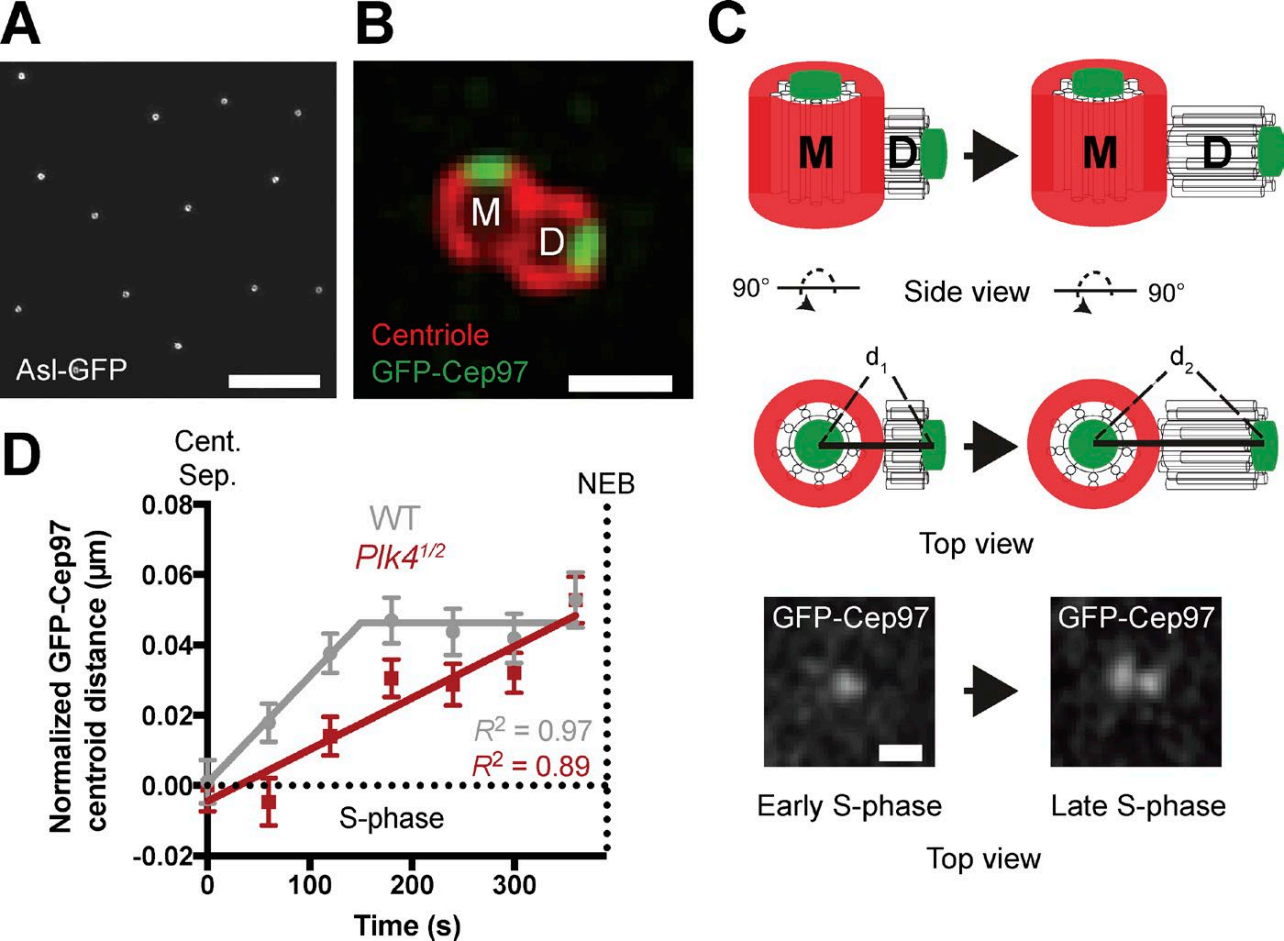
**A second assay to measure centriole growth using the centriole distal-end binding protein GFP-Cep97. (A)** Micrograph shows a 3D-SIM image of an embryo expressing Asl-GFP (a mother centriole marker), illustrating how mother centrioles in these embryos are preferentially oriented end-on to the cortex (so each mother centriole appears as a hollow ring; see also Fig. S5 and Video 5). Bar, 5 µm. **(B)** Micrograph shows a 3D-SIM image of a centriole pair in a fixed *D. melanogaster* spermatocyte expressing the distal-end-binding protein GFP-Cep97. These cells have unusually large centrioles (González et al., 1998), allowing one to easily distinguish the proximal and distal ends. The outer wall of the centriole is revealed by Asl-staining (red), which in spermatocytes is detected on both mother and daughter centrioles. GFP-Cep97 foci (green) are concentrated at the distal end of both the mother (M) and daughter (D) centriole. Bar, 0.5 µm. **(C)** Schematic shows how the distance between the GFP-Cep97 signal at the distal ends of the mother and daughter would be expected to increase as the centriole grows from early S-phase (d_1_) to late S-phase (d_2_). The schematic shows the centriole pair viewed both from the side and the top of the mother. The latter view resembles how the centrioles would usually be viewed in the early embryo, allowing us to measure daughter centriole growth in essentially 2D rather than 3D. Micrographs below show typical images of GFP-Cep97 in early and late S-phase, acquired on a Zeiss-880 Airyscan system. Bar, 0.25 µm. **(D)** Graph shows daughter centriole length over time, measured using this GFP-Cep97 assay in WT (*n* = 4 embryos; *n* = 69 centrioles in gray) and *Plk4^1/2^* embryos (*n* = 5 embryos; *n* = 84 centrioles in red). These daughter centriole growth profiles are similar to those obtained using the Sas-6-GFP incorporation assay (see Fig. 6 A). Note that centriolar GFP-Cep97 levels decrease toward the end of S-phase, so we cannot accurately track the GFP-Cep97 signal up to NEB. Data are shown as mean ± SEM; *R*^2^ is used as a measure for goodness-of-fit.

We wanted to confirm the kinetics of daughter centriole growth using an assay that was independent of Sas-6-GFP fluorescence incorporation. Our 3D-SIM analysis of the centrioles in early *D. melanogaster* embryos revealed that mother centrioles are usually oriented end-on to the cortex, so they appear as hollow rings (Fig. 7 A, Fig. S5, and Video 5). Thus, mother centrioles do not freely rotate in the *z*-axis, but rather adopt a relatively fixed orientation in reference to the cortex. We reasoned, therefore, that we could use the centriole distal-end binding protein GFP-Cep97 (Fig. 7 B) to measure the distance between the center of the mother centriole and the distal end of the growing daughter using Airyscan superresolution microscopy (Fig. 7 C).

We acquired superresolution images of embryos expressing GFP-Cep97, determined the center of mass (COM) of the mother and of the distal end of the growing daughter, and measured the distance between them as S-phase progressed (note that all scoring was performed blind; Fig. 7, C and D). This method produced centriole growth curves similar to the Sas-6-GFP incorporation assay in both WT and *Plk4^1/2^* embryos, confirming the kinetics of daughter centriole growth in WT embryos, and that daughter centrioles grow more slowly but for a longer period of time in *Plk4^1/2^* embryos, and so reach the same size as in WT embryos (Fig. 7 D).

### Sas-6 incorporates into the proximal end of growing daughter centrioles

**Figure 8.**
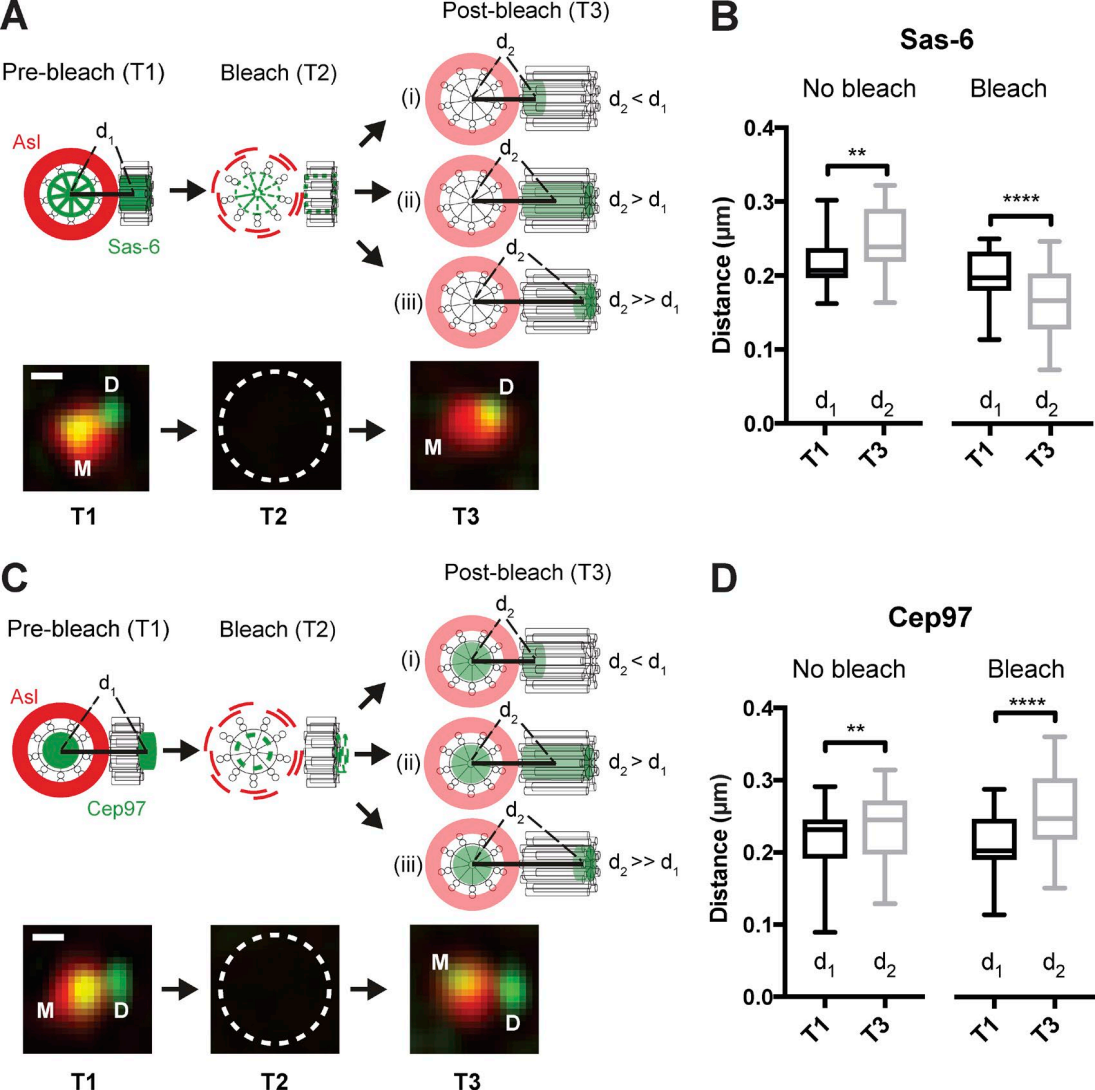
**Sas-6-GFP is incorporated into the proximal end of the growing daughter centriole. (A)** Experimental scheme to test the site of centriolar Sas-6-GFP incorporation. Embryos expressing Sas-6-GFP and Asl-mCherry were allowed to proceed through S-phase until daughter centrioles were approximately halfway through their growth period. The distance (d_1_) between the centroid of the mother (calculated using the Asl-mCherry signal) and daughter (calculated using the Sas-6-GFP signal) was measured (time = T1). The centriole pair was bleached (time = T2; note that bleaching the GFP fluorescence unintentionally also bleached the Asl-mCherry fluorescence, but Asl-mCherry fluorescence at the mother rapidly recovered), and a second image acquired 1 min later (time = T3), when the distance between the centroids was measured again (d_2_). The difference between d_2_ and d_1_ will depend upon the site of Sas-6-GFP incorporation, as illustrated. Micrographs below the schematic illustrate examples of the images acquired at the corresponding time points. Bar, 0.2 μm. **(B)** Graph quantifies d_1_ and d_2_: in unbleached centrioles, which have grown slightly between T1 and T3, d_2_ is greater than d_1_; after bleaching d_2_ is less than d_1_, indicating that Sas-6-GFP is incorporated proximally. *n* = 10 embryos; *n* = 23 centriole pairs for control (unbleached) and 24 pairs for FRAP. **(C and D)** These panels show similar data to that shown in A and B, but for a control experiment examining where the distal-end-binding protein GFP-Cep97 is incorporated. *n* = 10 embryos; *n* = 25 centriole pairs for control (unbleached) and 28 pairs for FRAP. In this control, GFP-Cep97 appears to be incorporated distally, as expected, indicating that these methods are sensitive enough to distinguish between the proximal incorporation of Sas-6-GFP (B) and the distal incorporation of GFP-Cep97 (D). Bar, 0.2 μm. Midlines represent the median, whiskers (error bars) mark the minimum to maximum, and bottom/top of the boxes indicate the first/third quartile of the distribution, respectively. Statistical significance was assessed using a paired *t* test (**, P < 0.01; ****, P < 0.0001).

Our observation that Plk4 influences the parameters of daughter centriole growth led us to ask whether the centriole cartwheel grows by incorporating Sas-6 at its proximal or distal end or isotropically throughout its length (Fig. 8 A). We generated embryos expressing Sas-6-GFP and Asl-mCherry (a marker of the mother centriole; Novak et al., 2014) and allowed daughters to grow to approximately half their full size. We then acquired a superresolution fluorescence image (Fig. 8 A, T1), and measured the distance between the COM of the mother and daughter centrioles (Fig. 8 A, distance d_1_). We photobleached the Sas-6-GFP (Fig. 8 A, T2)—which unintentionally also bleached the Asl-mCherry, but this fluorescence rapidly recovers (Novak et al., 2014)—allowing us to calculate the COM of the mother centriole at subsequent time points. We allowed the daughter centrioles to grow and incorporate Sas-6-GFP for 1min before acquiring a third superresolution image (Fig. 8 A, T3) and measuring the distance between the COM of the mother centriole and the newly incorporated Sas-6-GFP (Fig. 8 A, distance d_2_). This analysis revealed that in nonbleached controls d_2_ > d_1_, as expected, because the daughter centriole has grown in the time between T1 and T3. In bleached centrioles, however, d_2_ < d_1_, indicating that Sas-6-GFP is incorporated into the proximal end of the growing daughter (Fig. 8 B).

As an additional control, we used the same strategy to assess whether the centriole distal-end binding protein GFP-Cep97 incorporated into the distal end of the centriole after bleaching (Fig. 8, C and D); this was the case, confirming that our methods are sensitive enough to distinguish proximal- and distal-end incorporation. It is currently unclear how Sas-6-GFP can incorporate into the proximal end of the growing cartwheel while the daughter centriole maintains an “engaged” connection to its mother.

### Plk4 levels oscillate at the centriole

To better understand how Plk4 might influence the parameters of centriole growth, we quantified its centriolar localization during the embryonic nuclear cycles. Plk4 is usually present at very low levels in cells (Bauer et al., 2016), and neither the localization of the endogenous protein nor its detection by Western blotting has been reported previously in flies. We therefore generated flies expressing Plk4-GFP under the control of endogenous *Plk4* promoter in the *Plk4* mutant background, where it rescued the uncoordinated fly phenotype (Fig. S1 E and Video 6) and the centriole duplication defects (Fig. 5, D and E) observed in the *Plk4* mutant.

**Figure 9.**
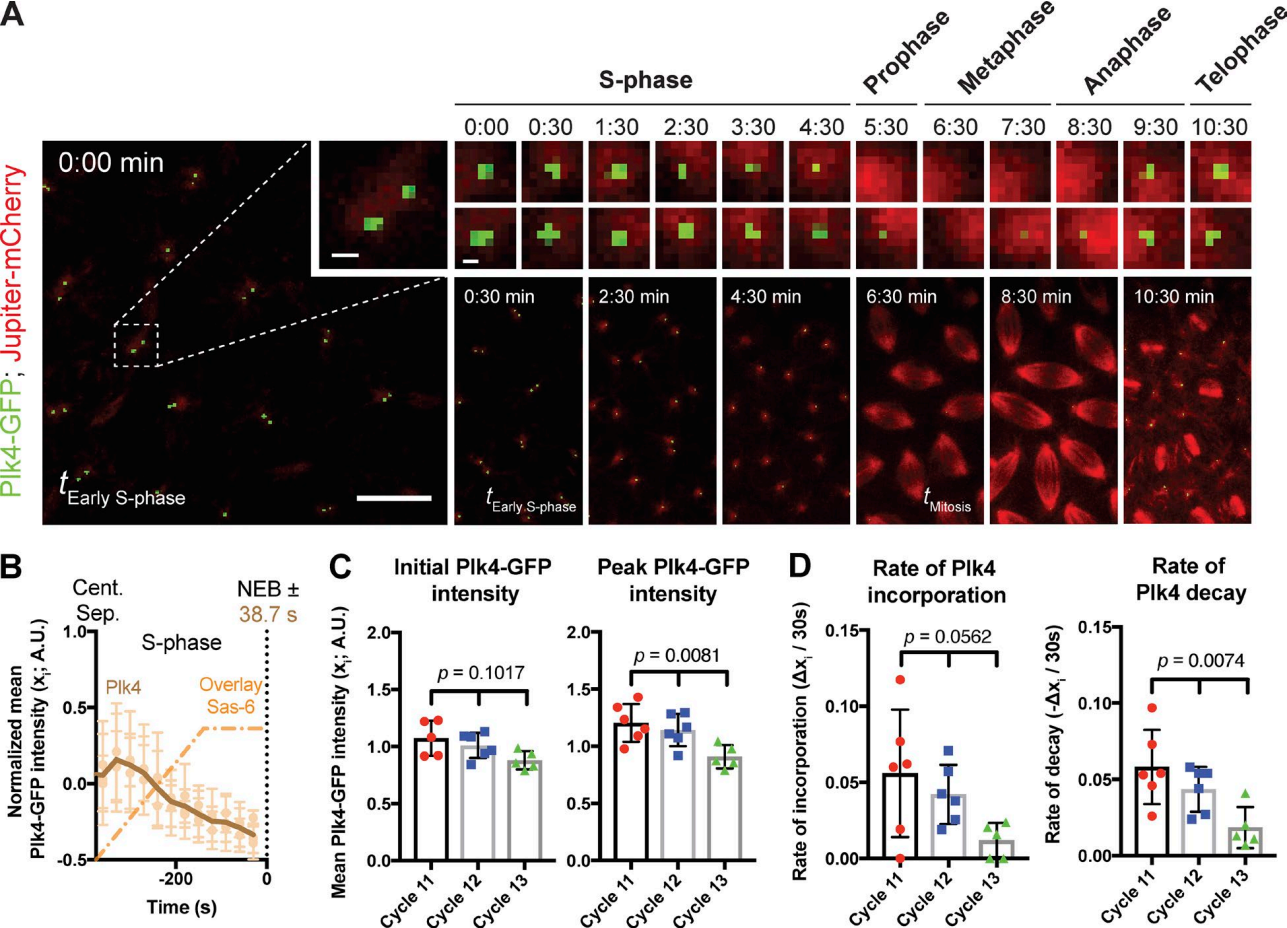
**An analysis of Plk4 centriolar dynamics. (A)** Micrographs show spinning-disk confocal fluorescence images taken from a time-lapse movie of an early embryo expressing Jupiter-mCherry (as a microtubule marker) and Plk4-GFP; time in minutes:seconds is indicated. At time *t* = 0:00, the embryo is in early S-phase, and the centrosomes have recently separated. Inset shows a single centriole pair (bar, 1 µm), which is then shown at 30-s or 1-min intervals (bar, 0.5 µm). The cell-cycle stage is indicated above each time interval. A series of lower-magnification views of the embryos are shown below the images of the individual centrioles to illustrate how MTs were used to determine the cell-cycle stage at each time point. Bar, 10 µm. **(B)** Graph shows the mean Plk4-GFP (brown) incorporation profile during S-phase of nuclear cycle 12 in WT embryos (*n* = 6 embryos). The mean incorporation profile of Sas-6-GFP is shown overlaid (dotted orange, same data shown in Fig. 3 A, but normalized for the length of S-phase). **(C and D)** Charts quantify parameters of Plk4-GFP behavior derived from mean Plk4-GFP profiles from embryos at nuclear cycles 11, 12, and 13 (*n* ≥ 5 embryos for each cycle; *n* = 34, 31, and 33 centriole pairs [mean] per embryo, respectively). A.U., arbitrary units. Data are represented as mean ± SD. Statistical significance was assessed using either an ordinary one-way ANOVA test (for Gaussian-distributed data) or a Kruskal–Wallis test, and is shown above each chart.

Centriolar Plk4-GFP levels oscillated in cycling early embryos: levels were lowest at metaphase, increased during late mitosis and early S-phase, and then abruptly started to decline in early-to-mid S-phase (Fig. 9, A and B; and Video 7). This decline was not caused by photobleaching, as the level of centriolar Plk4-GFP fluorescence started to increase again after metaphase of the next mitosis. As we cannot detect endogenous Plk4 by immunofluorescence, we cannot confirm that this localization reflects the localization of the endogenous Plk4. These mutant embryos that lack endogenous Plk4 but express Plk4-GFP can survive, however, demonstrating that Plk4-GFP can support the very rapid cycles of centriole duplication that are essential for early embryo development (Stevens et al., 2007; Varmark et al., 2007; Rodrigues-Martins et al., 2008).

From these data, we infer that daughter centrioles can grow at a constant rate even as centriolar Plk4-GFP levels fluctuate (Fig. 9 B). This suggests that not all of the Plk4-GFP recruited to centrioles during S-phase directly promotes daughter centriole growth (see Discussion). Interestingly, slightly lower levels of Plk4-GFP were recruited to centrioles at each successive nuclear cycle (Fig. 9 C), potentially explaining, at least in part, why daughter centrioles grow more slowly but for a longer period of time at successive nuclear cycles. Moreover, the centriolar levels of Plk4-GFP at the start of S-phase appeared to influence the rate at which Plk4-GFP was recruited to, and then lost from, the centrioles: in nuclear cycle 11, for example, centriolar Plk4 levels were relatively high, and the rate of Plk4 recruitment to, and subsequent loss from, the centrioles was also relatively high; during nuclear cycle 13, centriolar Plk4 levels were relatively low, and the rate of Plk4 recruitment to, and subsequent loss from, the centrioles was also relatively low (Fig. 9 D). These findings suggest that the levels of centriolar Plk4 at the start of S-phase can influence how fast Plk4 is subsequently recruited to, and then lost from, the centrioles.

## Discussion

**Figure 10.**
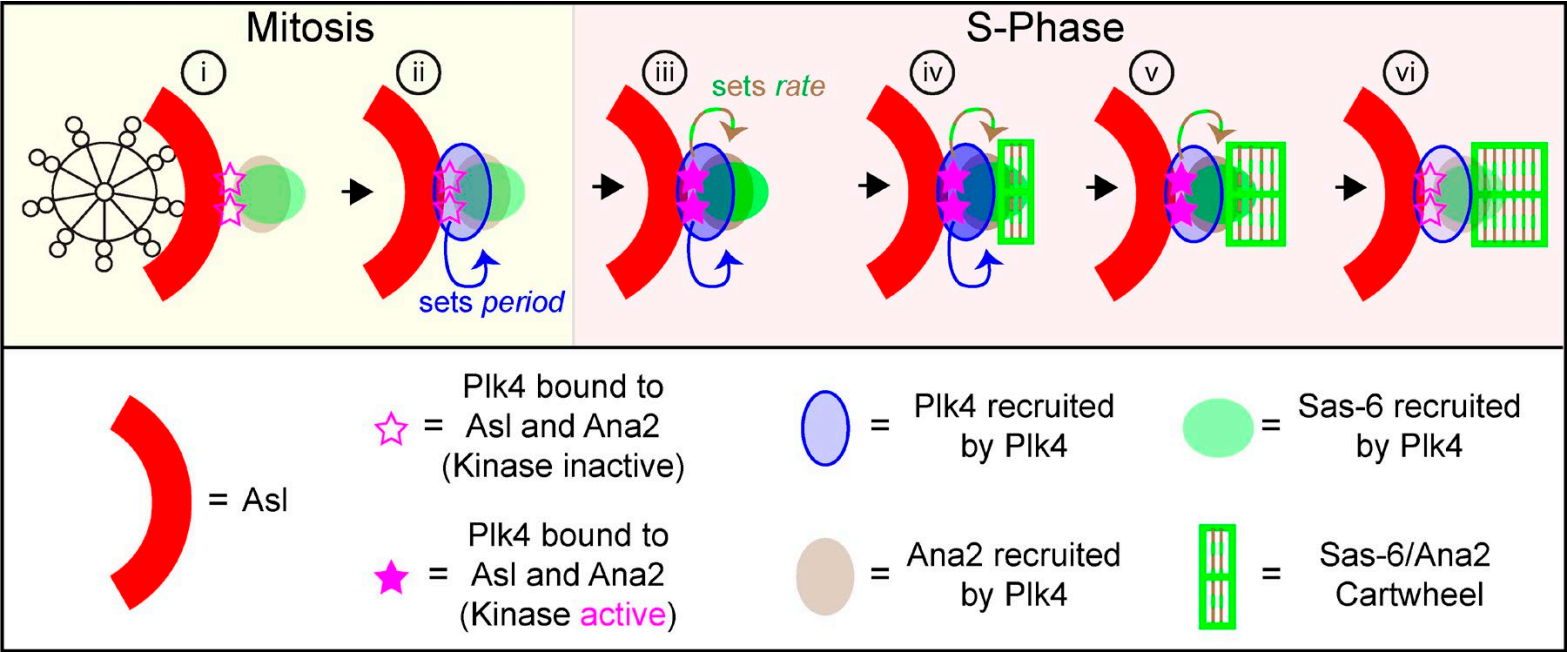
**Schematic illustration of how a Plk4-dependent homeostatic clock might set daughter centriole length in flies. (i)** The schematic shows an end-on view of a mother centriole (black skeleton) in mitosis, just after it has disengaged from its daughter. Plk4 starts to be recruited to mothers by the surrounding ring of Asl (red, only shown in part) but we speculate that a small fraction of Plk4 (pink star) is stabilized by binding to Ana2 (brown)—and potentially Sas-6 (green)—so defining the site where the daughter will form. **(ii)** This pool of Plk4 starts to recruit more Plk4 (blue arrow, recruiting to blue ellipse); the rate of this recruitment is dependent on the amount of Plk4 initially bound to Asl and Ana2, and it sets the period of daughter centriole growth by determining how quickly Plk4 will accumulate to trigger its own destruction. **(iii)** When the embryo enters S-phase, the Asl/Ana2-bound fraction of Plk4 is activated (filled pink stars), allowing it to recruit more Sas-6 and Ana2 (green/brown arrow); the kinase activity of the Plk4 influences the rate of Sas-6/Ana2 recruitment, and so the rate of centriole growth. **(iv)** Sas-6 and Ana2 levels reach a threshold that allows cartwheel assembly, whereas the local concentration of Plk4 continues to increase. **(v)** Centriolar Plk4 levels reach a critical concentration that triggers its destruction—centriolar Plk4 levels start to fall, but levels of the Asl/Ana2-bound Plk4 initially remain high enough that Sas-6 and Ana2 recruitment is not slowed, and cartwheel growth continues. **(vi)** Eventually, levels of the Asl/Ana2-bound Plk4 are too low to support Sas-6 and Ana2 recruitment, so their concentration falls below the threshold for cartwheel growth and the daughter centriole stops growing.

Several models have been proposed to explain how daughter centrioles might grow to the correct size (Goehring and Hyman, 2012; Marshall, 2015, 2016; Banterle and Gönczy, 2017), but none of these have been tested, primarily because of the lack of a quantitative description of centriole growth kinetics. Our observations suggest an unexpected, yet relatively simple, model by which centriolar Plk4 might determine daughter centriole length in flies (Fig. 10).

We propose that a small fraction of centriolar Plk4, perhaps the fraction bound to both Asl and Ana2 (Fig. 10, pink stars), influences both the rate of cartwheel growth (by determining the rate of Sas-6 and Ana2 recruitment to the centriole) and the period of cartwheel growth (by determining the rate of Plk4 recruitment to the centriole, and so how quickly centriolar Plk4 accumulates to trigger its own destruction). This model is consistent with our observation that daughter centrioles grow at a relatively constant rate even as centriolar levels of Plk4 fluctuate (indicating that the majority of Plk4 located at the centriole during S-phase is not directly promoting daughter centriole growth) and that centriolar Plk4 levels appear to influence the rate at which Plk4 is accumulated at centrioles (suggesting that Plk4 can recruit itself, either directly or indirectly, to centrioles).

In this model, Plk4 functions as a homeostatic clock, regulating both the rate and period of daughter centriole growth, and ensuring an inverse relationship between them: the more “active” the Plk4, the faster the daughters grow, but the faster Plk4 is recruited and so inactivated. The activity of this Plk4 fraction is probably a function of both the total amount of Plk4 in this fraction and its kinase activity. We speculate that this activity is determined before the start of S-phase by a complex web of interactions between Plk4, Ana2, Sas-6, and Asl that influence each other’s recruitment and stability and also, directly or indirectly, Plk4’s kinase activity (Ohta et al., 2014; Arquint et al., 2015; Klebba et al., 2015; Moyer et al., 2015). These interactions are likely to be regulated by external factors (such as the basic cell cycle machinery), allowing cells to set centriole growth parameters according to their needs. In cells with a G1 period, for example, Plk4 could be activated as cells progress from mitosis into G1, allowing the mother centriole to recruit an appropriate amount of Sas-6 and Ana2/STIL at this stage, which could then be incorporated into the cartwheel when cells enter S-phase. This could explain why in some somatic cells Plk4 levels appear to be higher during mitosis/G1 than in S-phase (Rogers et al., 2009; Sillibourne et al., 2010; Ohta et al., 2014), and why Plk4 kinase activity appears to be required primarily during G1, rather than S-phase (Zitouni et al., 2016).

This model can explain why halving the dose of Plk4 leads to a decrease in the growth rate and an increase in the growth period: halving the dose of Plk4 would be predicted to lower both the kinase activity of centriolar Plk4 (so slowing the growth rate) and the amount of centriolar Plk4 (so increasing the growth period). It can also potentially explain why doubling the dose of Plk4 might change the growth period without changing the growth rate: increasing the dose could lead to an increased rate of Plk4 recruitment (because of its increased cytoplasmic concentration), without increasing the amount or kinase activity of the Plk4 fraction bound to Asl or Ana2 (if these were already near saturation). Finally, it could explain why decreasing the kinase activity of Plk4 decreases the rate of growth without changing the growth period: the decrease in Plk4 kinase activity might affect the rate at which it recruits Ana2/Sas-6 without affecting the amount of centriolar Plk4, and so the rate at which Plk4 recruits itself to centrioles (Fig. 10).

Importantly, although the cartwheel extends throughout the entire length of the daughter centriole in worms and flies, this is not the case in vertebrates, where centrioles exhibit a second phase of growth during G2/M and the centriolar MTs grow to extend beyond the cartwheel (Kuriyama and Borisy, 1981; Chrétien et al., 1997). We suspect that the homeostatic clock mechanism we describe here may regulate the initial phase of centriole/cartwheel growth in all species, but the subsequent extension of the daughter centriole beyond the cartwheel that occurs in vertebrates will likely require a separate regulatory network.

To our knowledge, the concept of a homeostatic clock regulating organelle size has not been proposed previously. This mechanism is plausible for Plk4, because it can behave as a “suicide” kinase: the more active it is, the faster it will trigger its own inactivation (Rogers et al., 2009; Guderian et al., 2010; Holland et al., 2010; Cunha-Ferreira et al., 2013). This mechanism relies on delayed negative feedback, a principle that helps set both the circadian clock (Knippschild et al., 2005) and the somite segmentation clock (Lewis and Ozbudak, 2007). A similar mechanism might operate with other kinases that influence organelle biogenesis and whose activity accelerates their own inactivation, such as PKC, which regulates lysosome biogenesis (Feng and Hannun, 1998; Gould and Newton, 2008; Li et al., 2016). It will be interesting to determine whether homeostatic clock mechanisms that rely on delayed negative feedback could regulate organelle size more generally.

## Materials and methods

### *D. melanogaster* stocks and husbandry

The specific *D. melanogaster* stocks used in this study are listed in Table S1, and the lines generated and tested here are listed in Table S2. Sas-6-, Ana2-, and Plk4-GFP constructs were made by cloning the genetic region of *Sas-6*, *ana2*, and *PLK4*, respectively, from 2 kb upstream of the start codon up to (but excluding) the stop codon, into pDONR-Zeo vector (Thermo Fisher Scientific), which was then recombined with the pUAST-GFPCT vector (pTWG-1076; Drosophila Genomics Resource Centre) via Gateway technology (Thermo Fisher Scientific). To generate a Plk4 construct without any fluorescent tag, the endogenous Plk4 stop codon was introduced to the Plk4 pDONR (just described) by site-directed mutagenesis, using the Quikchange II XL mutagenesis kit (Agilent Technologies). To generate a Plk4 construct with reduced kinase activity (*Plk4^RKA^*), the L89G substitution (Holland et al., 2010; Zitouni et al., 2016) was introduced into the Plk4 coding sequence by site-directed mutagenesis, using the Quikchange II XL mutagenesis kit (Agilent Technologies). Both constructs were then recombined with the pUAS-G-empty vector (this study; details available on request). The primer sequences used in the Gateway cloning for each gene are listed in Table S3. The primers used to generate the Plk4-RKA construct were provided by L. Gartenmann (University of Oxford, Oxford, England, UK). Transgenic lines were generated by the Fly Facility in the Department of Genetics, University of Cambridge (Cambridge, England, UK).

The *Plk4^Aa74^* allele was generated by inducing an imprecise excision of the P-element P{GSV1}GS3043 (Kyoto Stock Center, Kyoto Institute of Technology) located 996 bp upstream of the *PLK4* start codon. The resulting 1,751-bp deletion (Fig. 5 A) removed the first third of the coding sequence, including all sequences encoding the kinase domain (Fig. 5 B). *Plk4^Aa74^* mutant flies were viable but uncoordinated, unlike the hypomorphic *Plk4^c06612^* flies that retained some coordination (Fig. 5 C and Video 4). The uncoordinated phenotype of *Plk4^Aa74^* mutant flies was rescued by the expression GFP-Plk4 under the control of the Ubq promoter and by the expression of Plk4-GFP under the control of its own endogenous promoter (Fig. 5 C and Video 6). *Plk4^Aa74^* mutant third-instar larval brains expressing GFP-PACT had no detectable centrioles, a phenotype that was rescued by the expression of both GFP-Plk4 (Fig. 5, D and E) and Plk4-GFP (not depicted) under the control of Ubq promoter or of its endogenous promoter, respectively, whereas the hypomorphic *Plk4^c06612^* allele had reduced centriole numbers. Thus, the *Plk4^Aa74^* allele appears to be a null or a very strong hypomorph.

Flies were maintained at 18°C or 25°C on Drosophila culture medium (0.77% agar, 6.9% maize, 0.8% soya, 1.4% yeast, 6.9% malt, 1.9% molasses, 0.5% propionic acid, 0.03% ortho-phosphoric acid, and 0.3% nipagin) in vials or bottles. For embryo collections, 25% cranberry-raspberry juice plates (2% sucrose and 1.8% agar with a drop of yeast suspension) were used. Embryos studied in our imaging experiments were 0–1-h collections at 25°C, which were then aged at 25°C for 45–60 min. Before imaging, embryos were dechorionated by hand, mounted on a strip of glue painted on a 35-mm glass-bottom Petri dish with 14 mm micro-well (MatTek), and were left to desiccate for 1 min at 25°C. After desiccation, the embryos were covered with Voltalef grade H10S oil (Arkema).

### Behavioral assays

#### Hatching experiments

To examine the quality of the embryonic development for various fly strains generated in this study, 0–1-h collected embryos were aged for 24 h, and the percentage of the embryos that had hatched out of their chorion was calculated. For this assay, six technical repeats were performed over several days.

#### Negative gravitaxis experiments

A standard negative gravitaxis assay (Ma and Jarman, 2011; Pratt et al., 2016) was used to assess the climbing reflexes of *Sas-6*, *ana2*, and *Plk4* null mutant flies rescued, respectively, with Sas-6-GFP, Ana2-GFP, and Plk4-GFP. In brief, three technical repeats of 1–4-d-old adult male flies (*n* = ∼15 for each technical repeat) were sharply tapped to the bottom of a cylinder, and the maximum distance climbed by individual flies within the first 10 s after tapping was measured. The distances were calculated using Fiji (ImageJ).

### Immunoblotting

0–3-h embryos were collected at 25°C. They were chemically dechorionated and fixed with methanol as previously described (Stevens et al., 2010). Fixed embryos were preserved overnight at 4°C and rehydrated by washing in PBT (PBS and 0.1% Triton X-100) three times for 15 min. Using a bright-field dissecting microscope (with 50× magnification), 40 embryos were selected in a volume of 20 µl PBT, and this was mixed with 20 µl of 2× SDS loading buffer. The sample was lysed at 95°C for 10 min, and 10 µl suspension/sample was loaded onto 3–8% Tris-acetate precast gels for SDS-PAGE (Thermo Fisher Scientific). Proteins were then transferred to nitrocellulose membrane for Western blotting. Membrane was blocked using 4% milk powder in PBS and 0.1% Tween 20 for 1 h. Primary antibodies used in this study are as follows: anti–Sas-6 (rabbit; Peel et al., 2007), anti-Ana2 (rabbit; Stevens et al., 2010), anti-Cnn (rabbit; Dobbelaere et al., 2008), and anti-GFP (mouse; Roche) used 1:500 in blocking solution. The incubation period for primary antibodies was 1 h. Secondary antibody used for immunoblotting: HRPO-linked anti-rabbit IgG and HRPO-linked anti-mouse IgG (both GE Healthcare) diluted 1:3,000 in blocking solution. The incubation period for secondary antibody was 45 min, after which membranes were washed with three changes of TBST (TBS and 0.1% Tween 20) for 45 min. Membranes were incubated in SuperSignal West Femto Maximum Sensitivity Substrate (Thermo Fisher Scientific; 1:1 mix, diluted 1:2, 1:6, 1:10, and 1:5 in water for Sas-6, Ana2, Cnn, and GFP, respectively). Finally, membranes were exposed to film using exposure times that ranged from <1 to 30 s.

### Immunofluorescence

#### Immunostaining on late pupal testes and larval brains

Late pupal testes from Ubq-GFP-CG3980 (GFP-Cep97) strain and the third instar larvae brains from GFP-PACT strain were dissected in PBS on a silicon dish and fixed by incubation in 4% formaldehyde in PBS for 20 min (for brains) or 30 min (for testes). As an additional step, fixed brains were transferred to a drop of 45% acetic acid for 15 s and then to a drop of 60% acetic acid for 3 min. This was followed by squashing the testes in between two coverslips of thicknesses 1 and 1.5 (silicon-dipped), which had been placed between two pieces of Whatman filter papers (GE Healthcare). In the case of brains, the squashing step was done using a 1.5-thickness coverslip and a slide. After squashing, the sample was snap-frozen in liquid nitrogen for 10 min. Once taken out of the liquid nitrogen, the 1-thickness coverslip was carefully removed from either the 1.5-thickness coverslip (for testes) or the slides (for brains) using a razor blade. The samples were incubated in 100% cold methanol at −20°C for 5 min (for brains) or 100% cold ethanol at −20°C for 15 min (for testes) and were transferred to a Petri dish containing PBST (1% Tween-20 [Sigma-Aldrich] in PBS) to be incubated for 10 min. For testes, this was followed by three washes in PBS for 5 min and by incubation in PBST containing 1:500 Guinea pig polyclonal anti-Asl primary antibody (Roque et al., 2012) overnight at 4°C in a humid chamber. The sample was then washed three times with PBS for 5 min and incubated in PBST containing 1:500 anti–guinea pig IgG Alexa Fluor 568 secondary antibody (Thermo Fisher Scientific) and 1:500 GFP-booster coupled to Atto 488 (ChromoTek) at room temperature for 3 h. For brains, the sample was stained for 10 min in Hoechst 33258 (Thermo Fisher Scientific). After washing three times in PBS for 15 min, the sample was mounted in mounting medium.

#### Immunostaining on embryos

For immunostaining on embryos, 0–2-h old *Oregon-R* WT samples were collected and dechorionated in 60% bleach for 2 min. This was followed by a wash in 0.05% Triton X-100 in distilled water. Embryos were then washed into small glass ampules containing 100% heptane and were shaken gently with 3% 0.5 M EGTA in pure methanol. Samples were stored in methanol at 4°C. Refrigerated samples were rehydrated by a wash in PBT (0.1% Triton X-100 in PBS) and blocked in 5% BSA (in PBS) for 1 h at room temperature. Blocking was followed by incubating the samples in 5% BSA (in PBS) containing 1:500 guinea pig anti-Asl (Roque et al., 2012) or 1:500 rabbit anti-PLP (Martinez-Campos et al., 2004) overnight at 4°C. Samples were then washed with PBT before 3-h incubation in 5% BSA (in PBS) containing 1:1,000 anti-guinea pig IgG Alexa Fluor 488 (Thermo Fisher Scientific) or 1:1,000 anti-rabbit IgG Alexa Fluor 594 (Thermo Fisher Scientific) secondary antibodies. After washing three times in PBT, each for 15 min, samples were mounted using Vectashield mounting medium containing DAPI (Vector Laboratories) onto microscopy slides with high-precision glass coverslips (CellPath).

### Image acquisition, processing, and analysis

#### 3D SIM

Living embryos were imaged at 21°C using a DeltaVision OMX V3 Blaze microscope (GE Healthcare). The system was equipped with a 60×/1.42-NA oil UPlanSApo objective (Olympus Corp.), 488- and 593-nm diode lasers, and Edge 5.5 sCMOS cameras (PCO). Spherical aberration was reduced by matching the refractive index of the immersion oil (1.514) to that of the embryos. 3D-SIM image stacks consisting of six slices at 0.125-µm intervals were acquired in five phases and from three angles per slice. The raw acquisition was reconstructed using softWoRx 6.1 (GE Healthcare) with a Wiener filter setting of 0.006 and channel-specific optical transfer function. For two-color 3D-SIM, images from green and red channels were registered with the alignment coordination information obtained from the calibrations using 0.2-µm-diameter TetraSpeck beads (Thermo Fisher Scientific) in the OMX Editor software. The SIM-Check plug-in in ImageJ (National Institutes of Health) was used to assess the quality of the SIM reconstructions (Ball et al., 2015).

To carry out the FRAP experiments in 3D-SIM, the software development kit (GE Healthcare) was used. Settings for the sequence of events needed for FRAP experiments were as follows: (1) acquisition of a single *z*-stack in 3D-SIM (Fig. 1, pre-bleach); (2) multispot photobleaching (by the OMX galvo scanner TIRF/photo kinetics module); (3) acquisition of the photobleached image (Fig. 1, bleach); and (4) acquisition of the time-lapse images in 3D-SIM (Fig. 1, post-bleach).

Assessment of the mother centriole orientation in early embryos (Fig. S5) was performed by visually scoring the percentage of mother centrioles that formed clear hollow rings when stained with anti-Asl or anti-PLP antibodies.

#### Spinning disk confocal microscopy

Living embryos were imaged at 21°C using a PerkinElmer ERS Spinning Disk confocal system on a Zeiss Axiovert 200M microscope. The system was equipped with a Plan-Apochromat 63×/1.4-NA oil DIC lens. 488- and 568-nm lasers were used to excite GFP and mCherry, respectively (using fast-sequential mode for GFP only, and emission discrimination mode for GFP and mCherry together). Confocal sections of 13 slices with 0.5-µm-thick intervals were collected every 30 s. Focus was occasionally readjusted in between the 30-s intervals.

In embryos expressing Jupiter-mCherry and Plk4-GFP in a Plk4^Aa74^ homozygous background (data presented in Fig. 9 A), the fluorescent signal was too faint to be properly visualized on the system. We therefore used a system equipped with an EM-CCD Andor iXon+ camera on a Nikon Eclipse TE200-E microscopy using a Plan-Apochromat 60×/1.42-NA oil DIC lens, controlled with Andor IQ2 software. Confocal sections of 17 slices with 0.5-µm-thick intervals were collected every 30 s at 21°C.

Postacquisition image processing was performed using Fiji (National Institutes of Health). Maximum-intensity projections of the images were first bleach-corrected with Fiji’s exponential fit algorithm, and then the backgrounds were subtracted using the subtract background function with a rolling ball radius of 10 pixels. Centrioles (Sas-6-GFP foci) were tracked using TrackMate (Tinevez et al., 2017), a plug-in of Fiji, with the following analysis settings: track spot diameter size of 1.1 µm, initial threshold of >0.02, and quality of >0.07. Centriole growth regression curves were made using Prism 7 (GraphPad Software), and the mathematical modeling was done using the nonlinear regression (curve fit) analysis function. Growth curves in S-phase and mitosis were modeled discontinuously, as we reasoned that these two phases of the cell cycle are two separate entities (when data were modeled continuously, there was no statistically significant difference between the two ways of modeling). For S-phase, the data were initially fitted against three different functions to assess the most suitable model: linearity (or linear growth followed by a plateau), one-phase association (parabola), and sigmoidal (Fig. S2); among these models, linearity (or linear growth followed by a plateau) best fit the data. Thus, the data were modeled using two separate functions for S-phase: (1) linear growth and (2) linear growth followed by a plateau (Fig. 2 C). The latter function is an in-house algorithm where a linear line is tested against having a point of inflection at any point in S-phase after which the growth is constant. The equation is described as follows, where *b* represents the first intercept that leads to the point of inflection (*x*_0_, *y*_1_) after *x* amount of time with a slope of *m*:

$$\begin{array}{l} y_{1}=m*x+b\\ y_{x0}=m*x_{0}+b\\ y_{2}=y_{x0}\\ y=\mathrm{if}\left(x<x_{0},\;y_{1},\;y_{2}\right). \end{array}$$

The only constraint applied to this equation was the requirement of the condition that *m* and *x*_0_ must be greater than 0. For mitosis, the data were fitted against two separate functions: (1) linear growth and (2) constant (Fig. 2 D). In both the S-phase and mitosis analysis, centrioles that come from a single embryo were reasoned as internal replicates, and thus the fitting was done based on considering only the mean *y* value of each time point (in contrast to considering each replicate *y* value as an individual point). To judge and control the quality and precision of regression (goodness-of-fit), we used the *R*^2^ and absolute sum-of-square values as well as applying the runs test. To compare the fits, the extra sum-of-squares *F* test was applied, and the appropriate fit was chosen by selecting the simpler model unless the p-value was <0.05.

To create the final regression model curves of Sas-6-GFP, the best-fit parameters (means of growth rate, growth period [*m* and *x*_0_, respectively, in the equations] and S-phase length; see Fig. 2 D for the visual definition of these parameters) were calculated and origin-adjusted against the time point, ∼1–1.5 min after where the centrosomes from the previous cycle had separated (this was the time point at which TrackMate’s tracking algorithm could detect the centrosome separation with the threshold parameters). Plk4-GFP data were too noisy to predict a meaningful regression model; however, the regression analysis using linear increase and decrease functions in Prism 7 (taking the peak intensity value in Plk4-GFP’s growth curves as the point of inflection) allowed us to compare the differences between the rates of incorporation and decay over successive cell cycles (Fig. 9). To create the final nonregression model curve of Plk4-GFP, the raw curves from multiple embryos were averaged and plotted (Fig. 9 B, bold brown). The mean intensity values and the final model curves for all the proteins were normalized to zero by bringing the initial mean intensity values (just after centrosome separation) down to zero and normalizing the rest of the data accordingly. In all of these imaging experiments, the timing of nuclear envelope breakdown (NEB) in individual embryos was easily inferred from the sum intensity projections of *z*-slices, except in the case of Plk4-GFP, where we had to use the simultaneous expression of Jupiter-mCherry to determine the timing of NEB (Fig. 9 A and Video 7).

#### Airyscan superresolution microscopy

Living embryos in nuclear cycle 12 or cycle 14 were imaged at 21°C using an inverted Zeiss 880 microscope fitted with an Airyscan detector. The system was equipped with Plan-Apochromat 63×/1.4-NA oil lens. 488-nm argon and 561-nm diode lasers were used to excite GFP and mCherry, respectively (sequential excitation of each wavelength was switched per line to ensure green and red channels were aligned). Sections of five slices with 0.2-µm-thick intervals were collected every 1 min with a zoom value of 24.6 pixels/µm. Focus was readjusted between the 1-min intervals. Images were Airy-processed in 3D with a strength value of “auto” (∼6) or 6.5.

To measure the distancing between GFP-Cep97 foci at the distal ends of mother and daughter centrioles, three serial stacks were rapidly acquired (over a period of ∼10 s) at 1-min intervals through nuclear cycle 12. Multiple stacks were acquired at each time point to allow us to select the image that gave the best resolution of the two GFP-Cep97 foci at each centriole pair at each time point (as this varied, perhaps because of “wobbling” of the centriole pair). After acquisition, images were analyzed using their maximum-intensity projections in Fiji (see Fig. 7 C for an example). The GFP foci were thresholded to the extent that the threshold encapsulated only the entire intensity mass of the foci for each centriole. After thresholding, the COM for each GFP-Cep97 focus was calculated, and then the distance between COM of the foci on the daughter and mother was measured. The regression curves for GFP-Cep97 distancing were made and quality-controlled using the same methodology described in Spinning disk confocal microscopy. Although this methodology produces a daughter centriole growth profile similar to that seen with our Sas-6-GFP incorporation assay, we note that the data are very noisy, and this methodology may not be sensitive enough to detect subtle changes in centriole length in different conditions. We also note that the data from WT and half-dose embryos were scored blind.

We also used the Airyscan superresolution microscope to probe the site of incorporation of Sas-6-GFP and GFP-Cep97 into daughter centrioles (Fig. 8). Embryos were identified as they exited mitosis of nuclear cycle 13 and daughter centrioles were allowed to grow for 5 min into cycle 14 (where daughter centriole growth takes 10.7 ± 0.8 min [*n* = 4 embryos] in S-phase). This allowed centrioles to grow to roughly half of their final size, before the centrioles were photobleached. The following settings were used in terms of the sequence of events needed for FRAP experiments: (1) acquisition of a single *z*-stack using airy-scan (Fig. 8, T1); (2) multispot serial photo-bleaching (Fig. 8, T2); and (3) acquisition of the photobleached and the time-lapse images in Airyscan (Fig. 8, T3). To measure the distance between the COM of the mother centriole and the incorporated Sas-6-GFP or GFP-Cep97 (Fig. 8, A and C, distances d_1_ and d_2_), the center of Asl-mCherry focus was taken as the reference center of mother centriole for both pre- and postbleaching time points (Fig. 8, T1 and T3, respectively).

### Quantification and statistical analysis

All the details for quantification, statistical tests, *n*, definitions of center, and dispersion and precision measures are described in the main text, relevant figure legends, or Materials and methods. Significance in statistical tests was defined by P < 0.05. To determine whether the data values came from a Gaussian distribution, D’Agostino–Pearson omnibus normality test was applied. Prism 7 was used for all the modeling and statistical analyses.

### Online supplemental material

Fig. S1 compares the embryonic expression levels of Sas-6- and Ana2-GFP to their endogenous counterparts and quantifies the ability of Sas-6-GFP, Ana2-GFP, and Plk4-GFP transgenes to rescue the uncoordinated phenotype of their respective mutants. Fig. S2 illustrates the regression models tested to find the best-fit regression type for Sas-6-GFP dynamics. Fig. S3 shows the Sas-6-GFP model curves fitted to data from 15 different embryos in cycle 12. Fig. S4 compares the embryo hatching rates of embryos in which the genetic dosage or the activity of Plk4 was altered. Fig. S5 shows immunofluorescence data and quantification illustrating how mother centrioles are preferentially oriented end-on to the cortex in early fly embryos. Videos 1, 2, 4, and 6 compare the behavior of *Sas-6*, *Ana2*, or *Plk4* mutant flies to mutant flies rescued with their respective GFP-fusions. Video 3 shows an example of a time-lapse video of an embryo in nuclear cycle 12 expressing Sas-6-GFP and the tracks generated by TrackMate that were used to quantify Sas-6-GFP fluorescence levels through the cycle. Video 5 is a time-lapse 3D-SIM movie of Asl-GFP in early fly embryos, illustrating how mother centrioles remain oriented end-on with respect to the cortex through time. Video 7 illustrates the oscillating behavior of Plk4-GFP in cycle 12 of early embryogenesis. Table S1 lists the source of the *D. melanogaster* strains used in this study. Table S2 lists the *D. melanogaster* strains used in this study. Table S3 lists the sequences of the oligonucleotides used in this study.

## Supplementary Material

Supplemental Materials (PDF)

Video 1

Video 2

Video 3

Video 4

Video 5

Video 6

Video 7
