# Supplementary material for: A homeostatic clock sets daughter centriole size in flies
Source: J Cell Biol. 2018 Apr 2;217(4):1233–48. doi: 10.1083/jcb.201801014 (PMC5881511; doi:10.1083/jcb.201801014)
Supplement: Supplemental Materials (PDF) [file JCB_201801014_sm.pdf]

Supplemental material

Aydogan et al., https://doi.org/10.1083/jcb.201801014

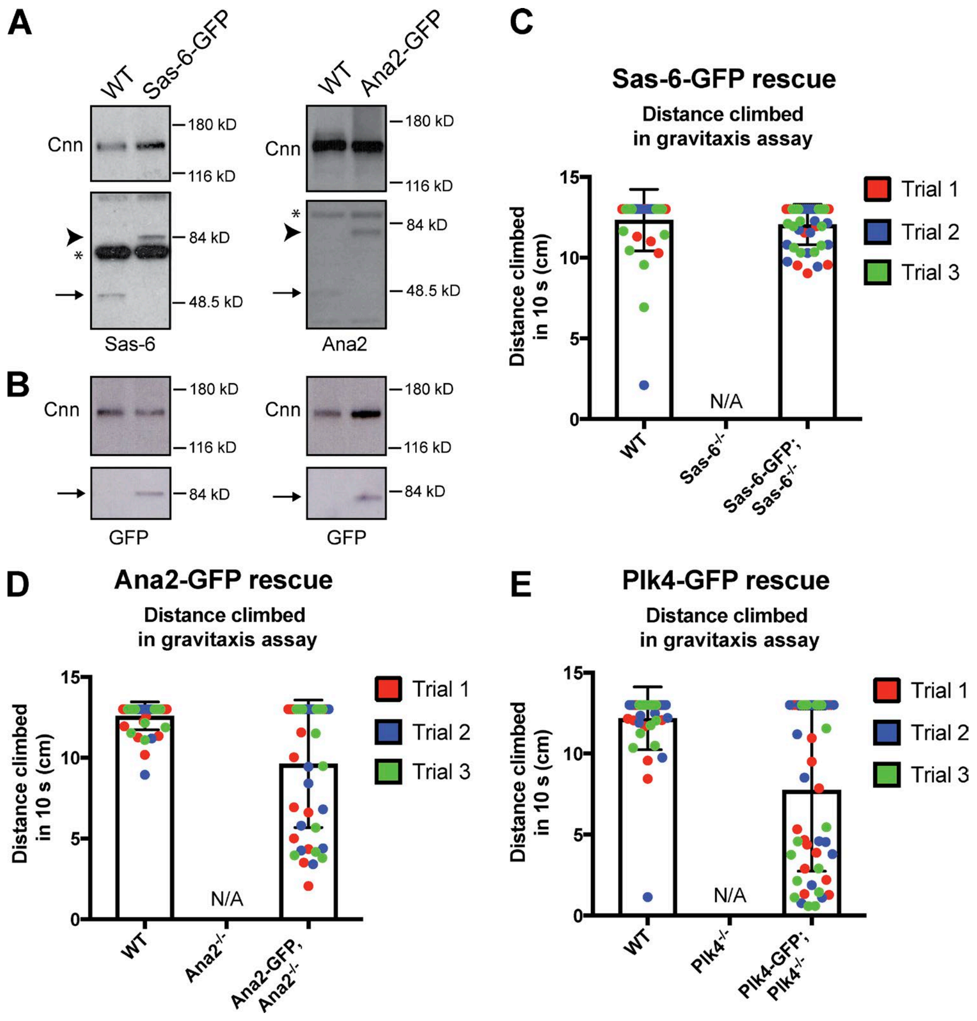

Figure S1. **Sas-6- and Ana2-GFP are expressed at similar levels as their endogenous proteins, and Sas-6-, Ana2-, and Plk4-GFP can at least partially rescue the uncoordinated phenotype of their respective adult mutant flies. (A)** Western blots show the protein levels of Sas-6-GFP (left) and Ana2-GFP (right; arrowheads) in transgenic embryos compared with their endogenous proteins in WT embryos (arrows). Cnn is shown as a loading control, and prominent nonspecific bands are indicated (*). **(B)** Similar blots were probed with anti-GFP antibodies to confirm that the GFP-fusion proteins were correctly identified. Cnn is shown as a loading control. Representative blots are shown from three technical repeats. **(C–E)** Graphs show the quantification of standard negative gravitaxis assays (Ma and Jarman, 2011; Pratt et al., 2016). In brief, three technical repeats of 1–4-d-old adult male flies (n ≈ 15 for each technical repeat) were sharply tapped to the bottom of a cylinder, and the maximum distance climbed by individual flies within the first 10 s after tapping was measured. Note that mutant flies were not scored in this assay, as it is difficult to keep them alive long enough to age them for the assay (as they are uncoordinated and have to be individually fed by hand to keep them alive). 100% of the mutant flies, however, were completely unable to climb (see Videos 1, 2, 4, and 6), so we show this bar as zero—marked with not/applicable (NA)—to give a reference of comparison for estimating the level of rescue for each transgene. Thus, although the Ana2-GFP and Plk4-GFP transgenes do not rescue the climbing activity of the mutant flies to the same level as WT flies, the level of rescue is appreciable. Data are presented as mean ± SD.

N = 15 embryos*

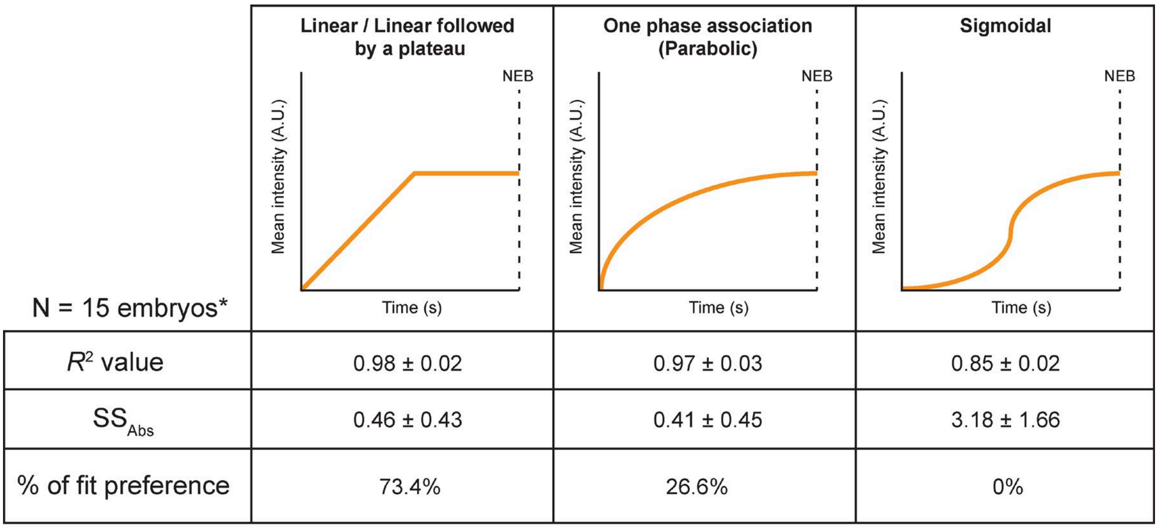

	Linear / Linear followed by a plateau	One phase association (Parabolic)	Sigmoidal
R^2 value	0.98 ± 0.02	0.97 ± 0.03	0.85 ± 0.02
SS_{Abs}	0.46 ± 0.43	0.41 ± 0.45	3.18 ± 1.66
% of fit preference	73.4%	26.6%	0%

*Reference data set: Cycle 12 in Figure 3

Figure S2. **The models tested in the regression analysis of Sas-6-GFP incorporation into the daughter centriole.** The Sas-6-GFP incorporation data for each individual embryo (see Fig. S3) was assessed using the nonlinear regression (curve fit) analysis function in GraphPad Prism 7. Growth curves in S-phase and mitosis were modeled discontinuously, as we reasoned that these two phases of the cell cycle are two separate entities. However, we also modeled the data continuously and found that there was no statistically significant difference between the two ways of modeling (not depicted). For S-phase, the data were initially fitted against three different functions (illustrated schematically in the graphs) to assess the most suitable model: linearity (or linear growth followed by a plateau), one-phase association (parabola), and sigmoid. Among these models, linear growth followed by a plateau was clearly the most preferable, and so all the data were fitted and analyzed with this model (as illustrated in Fig. S3). R^2 and absolute sum-of-squares values are used as a measure for goodness-of-fit. Sample size for modeling is indicated on the figure and in the legend of Fig. 3, from which the reference dataset (cycle 12) was obtained. A.U., arbitrary units.

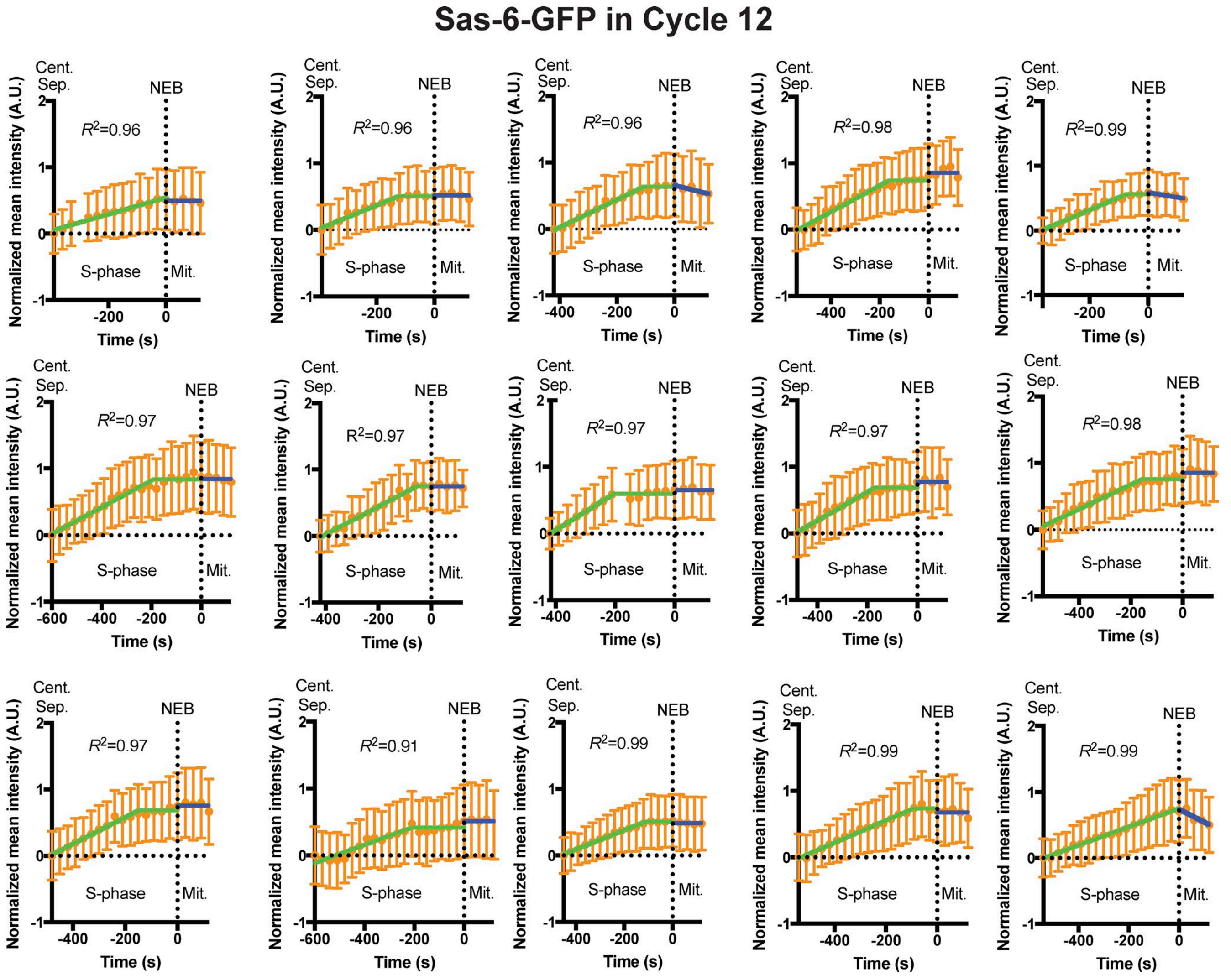

Figure S3. **Modeling Sas-6-GFP centriolar incorporation during nuclear cycle 12.** Graphs show the Sas-6-GFP incorporation profile from 15 different embryos at nuclear cycle 12. A regression analysis was performed independently on the S-phase (green line) and mitosis (blue line) data in each embryo using the model that best fit the overall data (linear growth followed by plateau, as described in Fig. S2). The R^2 value of the S-phase data is indicated in each graph. The full datasets for cycles 11 and 13 are not depicted. Note that, in a small number of embryos, Sas-6-GFP levels start to decrease as the embryos enter mitosis. We speculate that this is because in these embryos, some "excess" Sas-6-GFP might have been recruited to centrioles but is ultimately not incorporated into the cartwheel and then disperses back into the cytoplasm as the embryos enter mitosis. This finding is consistent with previous observations of SAS-6 dynamics in *Caenorhabditis elegans* embryos (Dammermann et al., 2008). A.U., arbitrary units. Data are presented as mean ± SD.

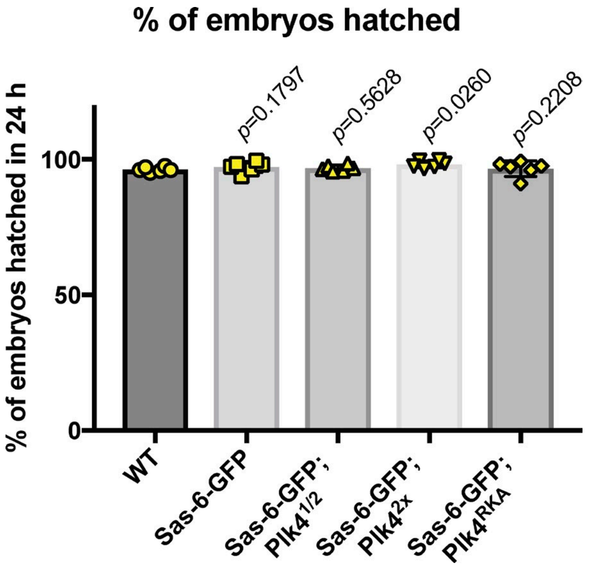

Figure S4. **Subtly altering the gene dosage or kinase activity of Plk4 does not detectably perturb embryo development.** Graph quantifies the hatching frequency of embryos laid by females of the indicated genotypes (which were all mated with WT males). The experimental lines expressed Sas-6-GFP (so we could measure the parameters of daughter centriole growth) and halved the genetic dose of *Plk4* ($Plk4^{1/2}$), doubled the dose of *Plk4* ($Plk4^{2X}$), or expressed a form of Plk4 with reduced kinase activity in an otherwise WT background ($Plk4^{RKA}$). None of these relatively subtle genetic changes detectably perturbed embryo development, as assessed by the hatching rate of the embryos. Data are represented as mean ± SD. Statistical significance was assessed using either an unpaired *t* test with Welch's correction (for Gaussian-distributed data) or an unpaired Mann–Whitney test, and is shown above each bar on the chart. n = 6 technical repeats for each group; per repeat n = 70 embryos in WT; n = 143 in Sas-6-GFP; n = 205 in Sas-6-GFP $Plk4^{1/2}$; n = 261 in Sas-6-GFP $Plk4^{2x}$; n = 193 in Sas-6-GFP $Plk4^{RKA}$.

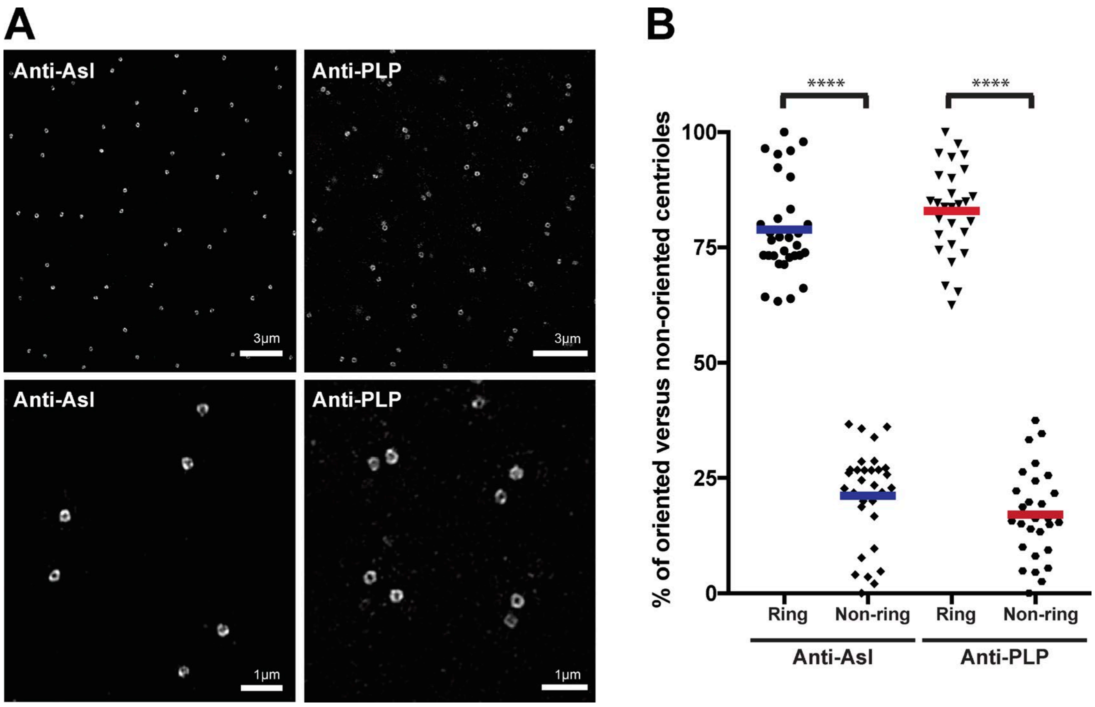

Figure S5. **Mother centrioles are preferentially oriented end-on to the embryo cortex. (A)** Micrographs show 3D-SIM images that illustrate how the majority of mother centrioles in fixed embryos are oriented end-on to the embryo cortex, so that they appear as hollow rings. The mother centrioles here were recognized with either anti-Asl (Roque et al., 2012) or anti-PLP (Martinez-Campos et al., 2004) antibodies. **(B)** Graph quantifies the percentage of centrioles that were clearly recognizable/not recognizable as rings with each antibody. Note that we believe this analysis of fixed embryos probably underestimates the percentage of centrioles that are orientated end-on to the cortex, because the antibody staining varied somewhat between embryos: in embryos that were not as brightly stained, the reduced signal/noise ratio made the SIM reconstructions less reliable, and so fewer centrioles could be unambiguously scored as rings. n = 31 and 28 embryos, respectively, for anti-Asl and anti-PLP staining; $n \geq 70$ centrioles (mean) per embryo. Statistical significance was assessed using a paired t test (****, P < 0.0001).

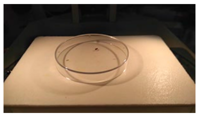

Video 1. ***Sas-6* mutant flies are uncoordinated, and this phenotype is rescued by the expression of Sas-6-GFP.** This montage shows short video clips of flies of the following genotypes: WT; *Sas-6* mutant (*Sas-6*$^{-/-}$); *Sas-6* mutant rescued by Sas-6-GFP flies (Sas-6-GFP; *Sas-6*$^{-/-}$). Like all flies that lack centrioles, the Sas-6 mutant is strongly uncoordinated because of the lack of cilia in its sensory neurons (Basto et al., 2006); this uncoordinated phenotype is strongly rescued by the expression of Sas-6-GFP. See also Fig. S1 C.

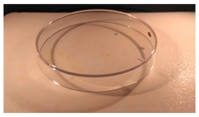

Video 2. ***Ana2* mutant flies are uncoordinated, and this phenotype is rescued by the expression of Ana2-GFP.** This video montage is similar to the one shown in Video 1 but shows the behavior of *Ana2* mutant flies and the rescue by Ana2-GFP. See also Fig. S1 D.

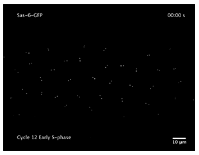

Video 3. **Monitoring Sas-6-GFP dynamics in early *D. melanogaster* embryos.** The first part of this video shows Sas-6-GFP foci followed through S-phase of nuclear cycle 12. The second part illustrates the dynamic tracking of Sas-6-GFP foci over time using the ImageJ plugin TrackMate. Note that TrackMate was set to generate "tracks" only for centriole pairs that were successfully monitored through every image of the S-phase period.

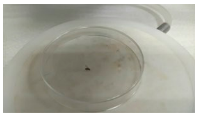

Video 4. **A comparison of the uncoordinated phenotype of the original *Plk4^{c06612}* mutant flies compared with the stronger *Plk4^{Aa74}* mutant flies we generate here.** This video montage shows short clips of flies of the following genotypes: the original *Plk4^{c06612}* mutation transheterozygous with a *Plk4* deficiency (*Plk4 hypomorph/Df*); the stronger *Plk4^{Aa74}* allele we generate in this study (*Plk4$^{-/-}$*); or the *Plk4^{Aa74}* mutant rescued by Plk4-GFP (Plk4-GFP; *Plk4$^{-/-}$*).

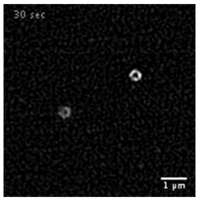

Video 5. **Mother centrioles are preferentially oriented end-on to the embryo cortex.** This short time-lapse video shows the behavior of the mother centriole marker Asl-GFP at two mother centrioles in mid-S-phase observed by 3D-SIM. Time in seconds is indicated. The centrioles are rapidly bleached over the 80-s time course of this video (with an image acquired every 10 s), so the images gradually degrade in quality as the movie proceeds; nevertheless, it can be seen that both centrioles maintain their end-on orientation relative to the embryo cortex.

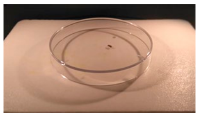

Video 6. ***Plk4* mutant flies are uncoordinated, and this phenotype is rescued by the expression of Plk4-GFP.** This video montage is similar to the one in Video 1 but shows the behavior of *Plk4* mutant flies and the rescue by Plk4-GFP. See also Fig. S1 E. Note that the video clip of the Plk4 mutant and the mutant rescued by Plk4-GFP are the same as those shown in Video 4.

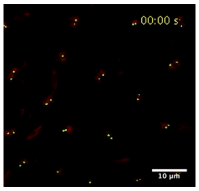

Video 7. **Centriolar Plk4-GFP levels fluctuate during the nuclear cycle.** This time-lapse video shows the behavior of Plk4-GFP (green) and Jupiter-mCherry (red), a marker of the microtubules, from early S-phase to late mitosis of nuclear cycle 12. The video was acquired on a spinning-disk confocal system.

Table S1. ***D. melanogaster* strains used in this study**

Allele[a]	Source	ID
Sas-6-GFP	This paper	NA
Sas-6^{c02901}	Peel et al., 2007	FlyBase ID: FBal0162156
Ana2-GFP	This paper	NA
ana2^{169}	Wang et al., 2011	FlyBase ID: Fbal0269126
ana2^{719}	Wang et al., 2011	FlyBase ID: Fbal0269127
Asl-mCherry	Conduit et al., 2015	NA
Ubq-GFP-CG3980 (GFP-Cep97)	Dobbelaere et al., 2008	NA
CycB^{2}	Jacobs et al., 1998	FlyBase ID: Fbal0094855
grp^{fsA4}	Sibon et al., 1997	FlyBase ID: Fbal0062815
Plk4^{Aa74}	This paper	NA
Plk4	This paper	NA
Plk4^{RKA}	This paper	NA
Asl-GFP	Blachon et al., 2008	FlyBase ID: FBtp0040947
asl^{B46}	Baumbach et al., 2015	NA
YFP-Asl	Varmark et al., 2007	FlyBase ID: FBtp0039668
Plk4-GFP	This paper	NA
Jupiter-mCherry	Callan et al., 2010	NA
Plk4^{c06612} (Hypomorph)	Bettencourt-Dias et al., 2005	FlyBase ID: FBal0159857
Plk4$^{Df(3L)Pc\text{-}2q}$ (Plk4^{Df})	Russell et al., 1996	FlyBase ID: FBab0022367
Ubq-GFP-Plk4	Basto et al., 2008	FlyBase ID: FBal0282567
Ubq-GFP-PACT	Martinez-Campos et al., 2004	FlyBase ID: FBal0190641

NA, not applicable.
[a]These alleles were expressed under their endogenous promoters unless ubiquitin expression is specified with the prefix *Ubq*.

Table S2. ***D. melanogaster* strains generated and/or used in this study**

**Strain genotype**	**Tissue used**	**Experiment (figure, video)**
Sas-6-GFP/Sas-6-GFP; Sas-6^{c02901}/Sas-6^{c02901}	Embryo	Figs. 1 A, 2 (B–D), 3, 4 (A and B), S1 (A–C), S2, and S3
Ana2-GFP, ana2^{169}/Ana2-GFP, ana2^{719}	Embryo	Figs. 1 B and S1 (A, B, and D)
Sas-6-GFP/Cyo	Embryo	Figs. 4 (C and D), 6, and S4
Sas-6-GFP/+; Plk4^{Aa74}/+	Embryo	Figs. 6 (A and B) and S4
Sas-6-GFP/Plk4; Plk4/+	Embryo	Figs. 6 (C and D) and S4
Sas-6-GFP/+; Plk4^{RKA}/+	Embryo	Figs. 6 (E and F) and S4
Asl-GFP/Asl-GFP; asl^{B46}/asl^{B46}	Embryo	Fig. 7 A
YFP-Asl/YFP-Asl	Embryo	Video 5
GFP-Cep97/+	Embryo; late pupal testis	Fig. 7 (B–D)
GFP-Cep97/+; Plk4^{Aa74}/+	Embryo	Fig. 7 D
Oregon-R (Wild-type)	Embryos; fly itself; third instar larval brain	Figs. 5 (B, D, and E), S1, S4, and S5; Videos 1, 2, 4, and 6
Asl-mCherry/Sas6-GFP	Embryo	Fig. 8 (A and B)
GFP-Cep97/+; Asl-mCherry/+	Embryo	Fig. 8 (C and D)
Plk4-GFP/+; Plk4^{Aa74}/Plk4^{Aa74}	Adult fly	Fig. S1 E; Videos 4 and 6
Plk4-GFP/+; Plk4^{Aa74}, Jupiter-mCherry/Plk4^{Aa74}	Embryo	Fig. 9; Video 7
Sas-6^{c02901}/Sas-6^{c02901}	Adult fly	Fig. S1 C; Video 1
ana2^{169}/ana2^{719}	Adult fly	Fig. S1 D; Video 2
Plk4^{Aa74}/Plk4^{Aa74}	Adult fly; third instar larval brain	Figs. 5 (B, C, and E) and S1 E; Videos 4 and 6
Asl-mCherry/Sas-6-GFP; Sas-6^{c02901}/Sas-6^{c02901}	Embryo	Fig. 1 C
Ana2-GFP, ana2^{169}/Ana2-GFP, ana2^{719}; Asl-mCherry/+	Embryo	Fig. 1 D
Sas-6-GFP/CycB^{2}	Embryo	Fig. 4 (C and D)
Sas-6-GFP/grp^{fsA4}	Embryo	Fig. 4 (C and D)
Plk4^{c06612}/Plk4^{c06612}	Adult fly; third instar larval brain	Fig. 5 (C and E)
Plk4^{c06612}/Plk4^{Df}	Adult fly	Video 4
Ubq-GFP-Plk4/+; Plk4^{Aa74}/Plk4^{Aa74}	Adult fly; third instar larval brain	Fig. 5 (C and E)
GFP-PACT/+	third instar larval brain	Fig. 5 D
GFP-PACT/+; Plk4^{Aa74}/+	third instar larval brain	Fig. 5 D

Table S3. **Oligonucleotides used in this study**

Oligonucleotide name	Sequence	Source
Primer for endogenous Plk4 (for GFP construct)	Forward: GCAGGCTTCAGACGACCTTTGGAGAACTGATTA	Thermo Fisher Scientific
Primer for endogenous Plk4 (for GFP construct)	Reverse: AGCTGGGTCAAGAAGCATGCGATTATAATAAGG	Thermo Fisher Scientific
Primer for endogenous Sas-6	Forward: GCAGGCTTCCTGTTTGATAACCACACAAAGCTTCGAAG	Thermo Fisher Scientific
Primer for endogenous Sas-6	Reverse: AGCTGGGTCTCGCCGATTTTCTTTGCCCGTG	Thermo Fisher Scientific
Primer for endogenous Ana2	Forward: GCAGGCTTCCTTTGTACAGGCTTCCCCATACGG	Thermo Fisher Scientific
Primer for endogenous Ana2	Reverse: AGCTGGGTCCAACAGCTTCGGCTGGTTCCTG	Thermo Fisher Scientific
Primer for $Plk4^{Aa74}$	Forward: TTGAAGTTCCGTTGGGTTGG	Thermo Fisher Scientific
Primer for $Plk4^{Aa74}$	Reverse: GCGAAAGTTCCAGGAAGTGG	Thermo Fisher Scientific
Primer to introduce stop codon into Plk4 pDONR by mutagenesis	Forward: CGCCTTATTATAATCGCATGCTTCTTTAAGACCCAGCTTTCTTGT ACAAAGTTGGC	Thermo Fisher Scientific
Primer to introduce stop codon into Plk4 pDONR by mutagenesis	Reverse: GCCAACTTTGTACAAGAAAGCTGGGTCTTAAAGAAGCATGCGATT ATAATAAGGCG	Thermo Fisher Scientific
Primer for $Plk4^{RKA}$	Forward: CAGCTCGAGCACCCCATACACATAGTTGGCGTCCTGAAAG	Thermo Fisher Scientific
Primer for $Plk4^{RKA}$	Reverse: CTTTCAGGACGCCAACTATGTGTATGGGGTGCTCGAGCTG	Thermo Fisher Scientific

References

Basto, R., J. Lau, T. Vinogradova, A. Gardiol, C.G. Woods, A. Khodjakov, and J.W. Raff. 2006. Flies without centrioles. *Cell*. 125:1375–1386. https://doi.org/10.1016/j.cell.2006.05.025

Basto, R., K. Brunk, T. Vinadogrova, N. Peel, A. Franz, A. Khodjakov, and J.W. Raff. 2008. Centrosome amplification can initiate tumorigenesis in flies. *Cell*. 133:1032–1042. https://doi.org/10.1016/j.cell.2008.05.039

Baumbach, J., Z.A. Novak, J.W. Raff, and A. Wainman. 2015. Dissecting the function and assembly of acentriolar microtubule organizing centers in Drosophila cells in vivo. *PLoS Genet*. 11:e1005261. https://doi.org/10.1371/journal.pgen.1005261

Bettencourt-Dias, M., A. Rodrigues-Martins, L. Carpenter, M. Riparbelli, L. Lehmann, M. Gatt, N. Carmo, F. Balloux, G. Callaini, and D. Glover. 2005. SAK/PLK4 is required for centriole duplication and flagella development. *Curr. Biol*. 15:2199–2207.

Blachon, S., J. Gopalakrishnan, Y. Omori, A. Polyanovsky, A. Church, D. Nicastro, J. Malicki, and T. Avidor-Reiss. 2008. Drosophila asterless and vertebrate Cep152 are orthologs essential for centriole duplication. *Genetics*. 180:2081–2094. https://doi.org/10.1534/genetics.108.095141

Callan, M.A., C. Cabernard, J. Heck, S. Luois, C.Q. Doe, and D.C. Zarnescu. 2010. Fragile X protein controls neural stem cell proliferation in the Drosophila brain. *Hum. Mol. Genet*. 19:3068–3079. https://doi.org/10.1093/hmg/ddq213

Conduit, P.T., A. Wainman, Z.A. Novak, T.T. Weil, and J.W. Raff. 2015. Re-examining the role of Drosophila Sas-4 in centrosome assembly using two-colour-3D-SIM FRAP. *eLife*. 4:1032. https://doi.org/10.7554/eLife.08483

Dammermann, A., P.S. Maddox, A. Desai, and K. Oegema. 2008. SAS-4 is recruited to a dynamic structure in newly forming centrioles that is stabilized by the γ-tubulin–mediated addition of centriolar microtubules. *J. Cell Biol*. 180:771–785. https://doi.org/10.1083/jcb.200709102

Dobbelaere, J., F. Josué, S. Suijkerbuijk, B. Baum, N. Tapon, and J. Raff. 2008. A genome-wide RNAi screen to dissect centriole duplication and centrosome maturation in Drosophila. *PLoS Biol*. 6:e224. https://doi.org/10.1371/journal.pbio.0060224

Jacobs, H.W., J.A. Knoblich, and C.F. Lehner. 1998. Drosophila Cyclin B3 is required for female fertility and is dispensable for mitosis like Cyclin B. *Genes Dev*. 12:3741–3751. https://doi.org/10.1101/gad.12.23.3741

Ma, L., and A.P. Jarman. 2011. Dilatory is a Drosophila protein related to AZI1 (CEP131) that is located at the ciliary base and required for cilium formation. *J. Cell Sci*. 124:2622–2630. https://doi.org/10.1242/jcs.084798

Martinez-Campos, M., R. Basto, J. Baker, M. Kernan, and J.W. Raff. 2004. The *Drosophila* pericentrin-like protein is essential for cilia/flagella function, but appears to be dispensable for mitosis. *J. Cell Biol*. 165:673–683. https://doi.org/10.1083/jcb.200402130

Peel, N., N.R. Stevens, R. Basto, and J.W. Raff. 2007. Overexpressing centriole-replication proteins in vivo induces centriole overduplication and de novo formation. *Curr. Biol*. 17:834–843.

Pratt, M.B., J.S. Titlow, I. Davis, A.R. Barker, H.R. Dawe, J.W. Raff, and H. Roque. 2016. Drosophila sensory cilia lacking MKS proteins exhibit striking defects in development but only subtle defects in adults. *J. Cell Sci*. 129:3732–3743. https://doi.org/10.1242/jcs.194621

Roque, H., A. Wainman, J. Richens, K. Kozyrska, A. Franz, and J.W. Raff. 2012. Drosophila Cep135/Bld10 maintains proper centriole structure but is dispensable for cartwheel formation. *J. Cell Sci*. 125:5881–5886. https://doi.org/10.1242/jcs.113506

Russell, S.R., G. Heimbeck, C.M. Goddard, A.T. Carpenter, and M. Ashburner. 1996. The Drosophila Eip78C gene is not vital but has a role in regulating chromosome puffs. *Genetics*. 144:159–170.

Sibon, O.C., V.A. Stevenson, and W.E. Theurkauf. 1997. DNA-replication checkpoint control at the Drosophila midblastula transition. *Nature*. 388:93–97. https://doi.org/10.1038/40439

Varmark, H., S. Llamazares, E. Rebollo, B. Lange, J. Reina, H. Schwarz, and C. González. 2007. Asterless is a centriolar protein required for centrosome function and embryo development in Drosophila. *Curr. Biol*. 17:1735–1745. https://doi.org/10.1016/j.cub.2007.09.031

Wang, C., S. Li, J. Januschke, F. Rossi, Y. Izumi, G. Garcia-Alvarez, S.S.L. Gwee, S.B. Soon, H.K. Sidhu, F. Yu, et al. 2011. An ana2/ctp/mud complex regulates spindle orientation in Drosophila neuroblasts. *Dev. Cell*. 21:520–533. https://doi.org/10.1016/j.devcel.2011.08.002
